# Seed dormancy loss from dry after-ripening is associated with increasing gibberellin hormone levels in *Arabidopsis thaliana*

**DOI:** 10.3389/fpls.2023.1145414

**Published:** 2023-05-18

**Authors:** Sven K. Nelson, Yuri Kanno, Mitsunori Seo, Camille M. Steber

**Affiliations:** ^1^ Molecular Plant Sciences Program, Washington State University, Pullman, WA, United States; ^2^ Plant and Data Science, Heliponix, LLC, Evansville, IN, United States; ^3^ Dormancy and Adaptation Research Unit, RIKEN Center for Sustainable Resource Science, Tsurumi, Yokohama, Japan; ^4^ Wheat Health, Genetics, and Quality Research Unit, USDA-ARS, Pullman, WA, United States; ^5^ Department of Crop and Soil Science, Washington State University, Pullman, WA, United States

**Keywords:** SLY1, Arabidopsis, dormancy, dry after-ripening, germination, seeds, gibberellin, abscisic acid

## Abstract

**Introduction:**

The seeds of many plants are dormant and unable to germinate at maturity, but gain the ability to germinate through after-ripening during dry storage. The hormone abscisic acid (ABA) stimulates seed dormancy, whereas gibberellin A (GA) stimulates dormancy loss and germination.

**Methods:**

To determine whether dry after-ripening alters the potential to accumulate ABA and GA, hormone levels were measured during an after-ripening time course in dry and imbibing ungerminated seeds of wildtype Landsberg *erecta* (L*er*) and of the highly dormant GA-insensitive mutant *sleepy1-2* (*sly1-2*).

**Results:**

The elevated *sly1-2* dormancy was associated with lower rather than higher ABA levels. L*er* germination increased with 2-4 weeks of after-ripening whereas *sly1-2* required 21 months to after-ripen. Increasing germination capacity with after-ripening was associated with increasing GA_4_ levels in imbibing *sly1-2* and wild-type L*er* seeds. During the same 12 hr imbibition period, after-ripening also resulted in increased ABA levels.

**Discussion:**

The decreased ABA levels with after-ripening in other studies occurred later in imbibition, just before germination. This suggests a model where GA acts first, stimulating germination before ABA levels decline, and ABA acts as the final checkpoint preventing germination until processes essential to survival, like DNA repair and activation of respiration, are completed. Overexpression of the GA receptor *GID1b* (*GA INSENSITIVE DWARF1b*) was associated with increased germination of *sly1-2* but decreased germination of wildtype L*er.* This reduction of L*er* germination was not associated with increased ABA levels. Apparently, *GID1b* is a positive regulator of germination in one context, but a negative regulator in the other.

## 1 Introduction

The regulation of seed germination is vital to plant and species survival. Seed dormancy prevents germination even under conditions suitable for germination in the short-term (Leubner-Metzger, 2005; Finch-Savage and Leubner-Metzger, 2006). Evolutionary benefits of seed dormancy include allowing time for seed dispersal, preventing germination out of season, and ensuring survival of natural disasters as seeds in the soil through a bet-hedging strategy (Koornneef et al., 2000; Venable, 2007; Poisot et al., 2011). Because of this, seed dormancy is more common in plants from areas with seasons (Baskin and Baskin, 1972). Dormancy can be lost through a period of dry storage called after-ripening. Depending on the species, dormancy can also be lost through scarification of the seed coat and water uptake in the cold called cold stratification. The precise control of physiological seed dormancy and dormancy loss are not fully understood, however the plant hormone abscisic acid (ABA) is known to promote dormancy whereas the hormone gibberellin A (GA) promotes seed germination (reviewed in Finkelstein et al., 2008). By examining changes in ABA and GA hormone levels at multiple after-ripening and imbibition timepoints, this study examined the timing of ABA and GA hormone changes in Arabidopsis seeds during after-ripening and imbibition before germination *per se*.

The process of seed germination in the strict sense begins with water uptake during imbibition and concludes with germination *per se*, the emergence of the radicle from the seed coat. The germination process involves the repair and activation of cellular systems needed for successful growth. The three phases of germination were defined by water uptake (reviewed in Bewley et al., 2013). Phase I involves the initial rapid uptake of water when the seed is first imbibed. Phase I processes include cellular rehydration, the initiation of DNA and mitochondrial repair, the translation of stored mRNAs, and the initiation of transcription. Dormancy may be imposed by repressors of these early cell processes. Seed moisture plateaus during Phase II. Phase II involves processes necessary for germination and post-germinative growth including: transcription and translation of new mRNAs, the completion of DNA repair, DNA synthesis, initial mobilization of stored reserves, and restoration of cellular integrity. The event of visible germination *per se* defines the end of Phase II and beginning of the post-germinative Phase III. The decision to germinate is likely tightly linked to regulation of Phase II processes since both dormant and non-dormant seeds enter Phase II, but only non-dormant seeds germinate (Nelson and Steber, 2017). Arabidopsis seeds do not germinate during cold stratification. However, cold stratification for short periods (3-5 d at 4°C) promotes germination, synchronizing seeds in early Phase II (Ogawa et al., 2003; Yamauchi et al., 2004; Cadman et al., 2006; Penfield and Springthorpe, 2012; Nelson and Steber, 2017). Longer cold stratification can induce secondary dormancy.

Dormancy loss involves a shift from dormancy-promotion by the sesquiterpene hormone ABA to germination-promotion by tetracyclic diterpene hormone GA. ABA levels peak during embryo maturation and remain high in mature dry seeds, consistent with its roles in establishment and maintenance of seed dormancy (Karssen et al., 1983; Lefebvre et al., 2006; Okamoto et al., 2006; Kanno et al., 2010; Lee et al., 2010). Mutants that reduce ABA biosynthesis or sensitivity cause decreased seed dormancy, while mutations in ABA turnover and hypersensitive mutants cause increased seed dormancy (reviewed in Finkelstein et al., 2002; Kushiro et al., 2004; Okamoto et al., 2006; Schramm et al., 2010; Schramm et al., 2013). Mutants that reduce GA biosynthesis or signaling increase seed dormancy or cause failure to germinate without exogenous GA application, whereas mutants with increased GA sensitivity are less dormant (Koornneef and van der Veen, 1980; Jacobsen and Olszewski, 1993; Steber et al., 1998; Olszewski et al., 2002; Willige et al., 2007). Furthermore, mature seed germination is inhibited by exogenous ABA application and stimulated by GA.

Several lines of evidence support the hormone balance theory suggestion that dormancy loss is correlated with a drop in endogenous ABA levels and a rise in GA levels (Karssen and Laçka, 1986). At timepoints late in imbibition, near the time of germination, ABA levels were higher in dormant than non-dormant Arabidopsis wild-type seeds, consistent with the idea that dormancy loss is associated with a decrease in ABA levels (Ali-Rachedi et al., 2004; Lee et al., 2010). Thus, dormancy appears to be associated with higher ABA levels during imbibition. Conversely, GA levels are very low in dry seeds and rise as seeds germinate (Jacobsen et al., 2002; Ogawa et al., 2003; Kanno et al., 2010). It is yet unclear, however, whether GA levels rise in response to dormancy loss before or after germination *per se*.

Detection of increasing bioactive GA prior to germination has been difficult because endogenous GA levels are much lower than ABA levels. In barley, an increase in bioactive GA_1_ was detected in after-ripened vs dormant embryos just as grains germinated (Jacobsen et al., 2002; Liu et al., 2013). Multiple imbibition timepoints from dry to 48 h imbibed barley indicated an increase in GA precursors with after-ripening that was strongest later in imbibition. However, some increase in GA biosynthetic precursors was also detected with after-ripening of dry seeds. To our knowledge, the only Arabidopsis data showing an increase in GA with after-ripening used the highly dormant GA-insensitive *sly1-2* (*sleepy1-2*) mutant (Ariizumi et al., 2013). In that study, a decrease in ABA and increase in GA levels was seen with long after-ripening (19 months) following cold stratification for 4 days in the dark plus 12 h imbibition in the light at 22°C (late Phase II). Using a modification of the sensitive GA-optimized method (Varbanova et al., 2007) for measuring hormones, this study measured GA levels in dry seeds and in imbibing seeds before germination to determine if after-ripening increases germination potential through an increase in bioactive GA levels.

Mutations in genes encoding GA biosynthetic enzymes established that GAs stimulate seed germination, stem and cell elongation, fertility, and flowering time (reviewed in Sun and Gubler, 2004; Ueguchi-Tanaka et al., 2007b; Yamaguchi, 2008). While over 136 gibberellins have been identified, only a subset are bioactive including GA_1_, GA_3_, GA_4_, and GA_7_ (Hedden, 2020). GA_1_ is the major bioactive GA in monocots like rice and barley, whereas GA_4_ is the major bioactive GA in dicots like Arabidopsis (Ueguchi-Tanaka et al., 2007a). The GA biosynthetic pathway begins with synthesis of *ent*-kaurene from geranylgeranyl diphosphate in the plastid (**Supplementary Figure 1**; reviewed in Yamaguchi, 2008; Hedden, 2016). This leads to phenotypes that are rescued by GA application including failure to germinate, extreme dwarfism, and failure to transition to flowering. GA_12_ is synthesized in the endoplasmic reticulum and serves as the common precursor for both GA_1_ and GA_4_. In Arabidopsis, mutations in enzymes acting prior to synthesis of GA_12_ result in failure to germinate and severe dwarfism. The *ga1-3* mutant largely blocks GA biosynthesis as a result of a deletion of the single copy of the early biosynthesis enzyme *ent*-copalyl diphosphate synthase (*CPS)* (Zeevaart and Talon, 1992). GA_1_ derives from hydroxylation of C-13 in GA_12_ to obtain GA_53_, whereas GA_4_ is derived from the non-13-hydroxylated GA_15_. The parallel non-13-hydroxylated and 13-hydroxylated synthesis of bioactive GA_4_ and GA_1_ are catalyzed by the P450 enzymes GA 20-oxidase (GA20ox) and GA 3-oxidase (GA3ox). Deactivation of bioactive GAs and their immediate precursors are catalyzed by GA 2-oxidase (GA2ox) activity. Mutations in *GA20ox* and *GA3ox* genes result in semi-dwarfs that are able to germinate without addition of GA, likely because they belong to multigene families. In Arabidopsis, there are five GA 20-oxidase genes, *GA20ox1* to *GA20ox5*, four GA 3-oxidase genes, *GA3ox1* to *GA3ox4*, and seven GA 2-oxidase genes, *GA2ox1* to *GA2ox8* (excluding *GA2ox5*, which is a pseudogene). Regulation of germination appears to largely depend on these late GA biosynthetic and turnover enzymes.

GA acts by lifting negative regulation GA responses by the DELLA (Asp-Glu-Leu-Leu-Ala) domain proteins (reviewed in Hauvermale et al., 2012). The absence of GA in *ga1-3* seeds causes high levels of DELLA protein to repress germination. GA treatment of *ga1-3* seeds triggers DELLA proteolysis and seed germination (Ariizumi and Steber, 2007). GA stimulates DELLA degradation through interaction with the GA receptor, GID1 (GA-INSENSITIVE DWARF1). In Arabidopsis there are three *GID1* GA-receptor genes, *GID1a*, *GID1b*, and *GID1c* (Nakajima et al., 2006). GA binding by GID1 causes a conformational change that exposes DELLA-interacting residues, leading to formation of a GID1-GA-DELLA complex (Murase et al., 2008; Shimada et al., 2008; Ueguchi-Tanaka et al., 2008; Hirano et al., 2010). Formation of the GID1-GA-DELLA complex causes the SLEEPY1 (SLY1) F-box subunit of an SCF (Skp, Cullin, F-box) E3 ubiquitin ligase to bind to DELLA (Steber et al., 1998; McGinnis et al., 2003; Dill et al., 2004). SCF^SLY1^ catalyzes DELLA polyubiquitination and proteolysis by the 26S proteasome. DELLA destruction alleviates negative regulation of GA responses including seed germination.

DELLA proteins are nuclear-localized proteins believed to regulate GA responses through interaction with transcriptional regulators including INDETERMINATE DOMAIN (IDD) and PHYTOCRHOME-INTERACTING FACTORS PIF1, 3, and 4 (Silverstone et al., 1998; Itoh et al., 2002; Zentella et al., 2007; Bai et al., 2012; Hirano et al., 2012; Yoshida et al., 2014; Zhiponova et al., 2014). A limited number of DELLA-activated gene promoters have been identified such as the activator of GA responses *SCARECROW-LIKE3* (*SCL3*), the GA biosynthesis enzymes *GA20ox2* and *GA3ox1*, the GA receptors *GID1a* and *GID1b*, and the *XERICO* positive regulator of ABA biosynthesis (Zentella et al., 2007; Yoshida et al., 2014). Since DELLAs are negative regulators of GA responses, DELLA activation of GA biosynthesis and signaling genes are likely negative feedback mechanisms. However, *XERICO* is a putative E3 ubiquitin ligase that positively regulates ABA biosynthesis (Piskurewicz et al., 2008). DELLA activation of *XERICO* transcription may indicate that GA stimulates germination by reducing XERICO-stimulation of ABA biosynthesis.

In Arabidopsis, mutations in positive regulators of GA signaling lead to increased seed dormancy and the failure to germinate associated with overaccumulation of DELLA proteins. Both mutations in the single *SLY1* F-box gene and the *gid1a gid1b gid1c* triple mutants result in a failure to germinate that cannot be rescued by GA (McGinnis et al., 2003; Griffiths et al., 2006; Ariizumi and Steber, 2007; Willige et al., 2007). Indeed, GA-insensitive mutants over-accumulate GA, likely as a feedback response to reduced signaling (Griffiths et al., 2006; Ariizumi et al., 2013). Mutations in *SLY1* do not entirely block GA signaling due to a mechanism of GA signaling that does not require DELLA proteolysis but does require GA and GID1 protein (McGinnis et al., 2003; Ariizumi and Steber, 2007; Hauvermale et al., 2022). GA-insensitive *sly1* mutants have elevated DELLA protein levels, a semi-dwarf stature, reduced male fertility, and increased seed dormancy. This study used the *sly1-2* frame-shift mutation resulting in loss of the last 40 amino acids (26% of the protein), including the proposed DELLA binding site. *sly1-2* seeds have a high degree of seed dormancy, requiring 1-3 years of after-ripening to reach 30-80% germination, whereas wild-type Landsberg *erecta* (L*er* wt) germinates efficiently with 2 weeks of after-ripening. The *sly1-2* germination phenotype is rescued both by long after-ripening and by overexpression of the *GID1* receptor genes. However, neither of these rescue mechanisms result in reduced DELLA protein accumulation. Thus, examination of hormonal changes in after-ripening *sly1-2* mutants will show changes that can occur without DELLA destruction. *GID1a*, *GID1b*, and *GID1c* overexpression (OE) all resulted in rescue of *sly1-2* seed germination, but rescue by *GID1b-OE* was strongest (Ariizumi et al., 2013). This is likely due to the fact that only GID1b can bind DELLA to some degree in the absence of GA, and that GID1b has higher affinity for GA and DELLA than either GID1a or GID1c (Griffiths et al., 2006; Nakajima et al., 2006; Yamamoto et al., 2010; Nelson and Steber, 2016). In addition to germination, *GID1* overexpression also caused partial rescue of other GA-regulated *sly1* phenotypes including plant height and fertility (Ariizumi et al., 2008; Ariizumi et al., 2013). Increasing GA sensitivity is likely a fundamental mechanism associated with dormancy loss since after-ripening is associated with increased expression of both *SLY1* mRNA and GID1 proteins (Lee et al., 2010; Hauvermale et al., 2015). It is possible that a relatively small increase in GA hormone level can stimulate germination as GA sensitivity increases with after-ripening or *GID1* overexpression. This study examined the changes in GA and ABA hormone levels associated with *sly1-2* germination-rescue by both long after-ripening and *GID1b* overexpression.

Seed dormancy is controlled through the balance between ABA and GA signaling. Many studies have examined changes in ABA levels with after-ripening, but few have investigated the corresponding changes in GA. This study examined the effects of after-ripening and *GID1b-OE* on wild-type and *sly1-2* ABA and GA hormone levels. In a previous study, we examined ABA and GA levels in *sly1-2* seeds at two after-ripening timepoints and an imbibition timepoint late in Phase II (Ariizumi et al., 2013). By taking a multidimensional approach to compare hormone levels across multiple after-ripening and imbibition timepoints, it was possible to ask novel questions about the changes in hormone levels associated with after-ripening and determine when in imbibition these changes can be observed. Furthermore, we demonstrated that loss of dormancy through after-ripening regulates the ability of cold stratification to stimulate an increase in GA during imbibition. By using a modification of the GA-optimized method of Plackett et al. (2012) for measuring hormone levels, we were able to detect GA in dry seeds and before germination across an after-ripening time course. Thus, we were able to show that GA hormone levels increase with after-ripening at a time in imbibition when ABA levels are high. This suggests that the role of GA in dormancy loss may precede that of ABA. We showed that *GID1b-OE* in wild-type resulted in decreased rather than increased germination as it did in *sly1-2*. Finally, we learned that the partial rescue for *sly1-2* germination by after-ripening and *GID1b-OE* is not associated with a decrease in ABA levels, suggesting that other mechanisms result in dormancy-release.

## 2 Materials and methods

### 2.1 Plant materials and growth conditions

*Arabidopsis thaliana* ecotype Landsberg *erecta* (L*er*) wild-type (wt), L*er GID1b-OE, sly1-2*, and *sly1-2 GID1b-OE* in the L*er* background were previously described (Steber et al., 1998; Ariizumi et al., 2008; Ariizumi et al., 2013). The *GID1b-OE* allele is a translational fusion of *HA : GID1b* expressed on the 35S cauliflower mosaic virus promoter. L*er* and *sly1-2* contain independent single copy, homozygous transformation events that were selected for similar expression levels by RT-qPCR analysis. All plants used in this study were grown side-by-side in the same Conviron^®^ growth chamber under fluorescent lights (200 µmol/m^2^/sec) according to McGinnis et al. (2003). The only exception to this was 21 month after-ripened (Long AR) *sly1-2*, which was grown under the same conditions in advance of the other seeds to allow capture of non-dormant *sly1-2* seeds in our experiments. Only the Long AR *sly1-2* seed batch was different from the batch used for 0, 2, and 4 week after-ripened *sly1-2* seeds timepoints. All other hormone measurements for the same Arabidopsis genotype came from the same original seed batch, differing only by length of after-ripening and/or time of imbibition when seeds were collected for hormone measurements.

Seeds for hormone measurements were harvested at “physiological maturity” to obtain a dormant seed starting point for all lines including wild-type L*er*, which is dormant at physiological maturity but loses dormancy rapidly through dry after-ripening (Russell et al., 2000). If Arabidopsis seeds are harvested after the plant has fully senesced and dried down, many seeds may have dry after-ripened for a few weeks since some siliques mature before others (Nelson et al., 2017). To obtain higher dormancy, L*er* seeds were harvested when plants were partially yellow and partially green (**Supplementary Figure 2A**). Mature, yellow siliques shatter and release seeds readily whereas green siliques do not. Collection of only mature, brown, dry seeds was achieved by harvesting only seeds that freely fell from siliques. **Supplementary Figure 2B** shows that only brown, physiological mature seeds were collected. No green seeds were used in the experiments. After harvest, seeds were stored at room temperature and low humidity (≈15-30%) in open tubes for the specified period of dry after-ripening, then stored in a -20°C freezer to maintain dormancy status until use. Dormant 0 day/week after-ripened (0wkAR) seeds were stored overnight in open tubes. After-ripening timepoints used in this study were defined by additional storage for 1 d (1dAR), 2 d (2dAR), 2 weeks (2wkAR), 4 weeks (4wkAR), 5 weeks (5wkAR), or 21 months (Long AR).

### 2.2 Germination experiments

**Figure 1:**
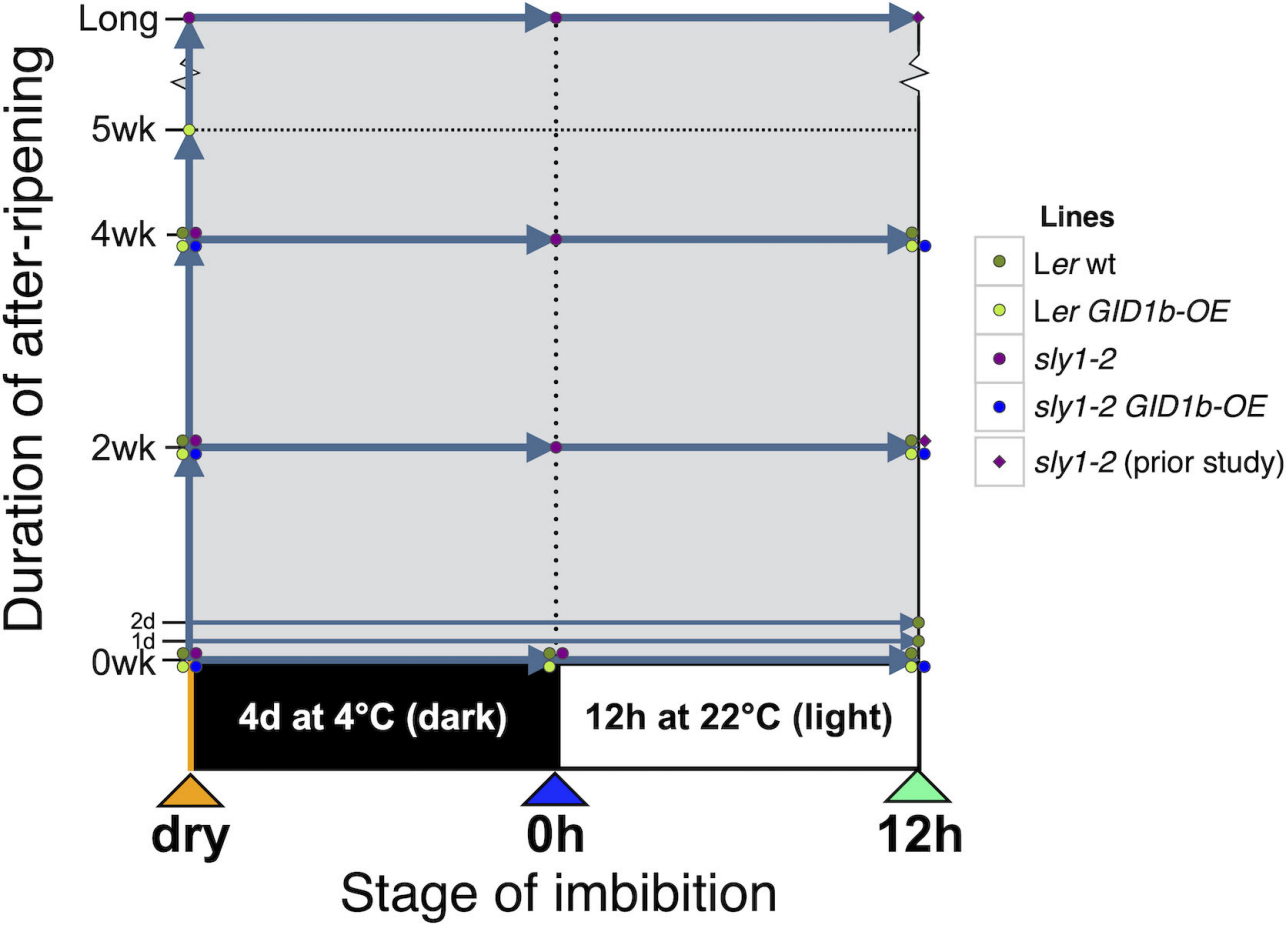
Diagram of sample timepoints in two dimensions: after-ripening (AR) and imbibition. Seeds are first stored for the specific duration of after-ripening and then samples were collected at the specified point in imbibition. After-ripening timepoints include: 0 days/weeks (d is day, wk is week), 1 d, 2 d, 2 wk, 4 wk, 5 wk, and long-after-ripened *sly1-2* stored for 21 months (mo is months). Imbibition timepoints include: dry seed before the start of imbibition, “0h” seeds imbibed for 4 days at 4°C in the dark, and “12h” seeds imbibed for 4 days at 4°C and then moved to the light at 22°C for 12 hours. Data for *sly1-2* at the 12h imbibition timepoint with 2 weeks or long after-ripening are from Ariizumi et al. (2013).

All germination screens were conducted with three replications of 60 to 100 seeds per replicate. Seeds were sterilized for 5 minutes in 70% ethanol and 0.01% SDS, followed by 10 minutes in 10% bleach and 0.01% SDS, washed thoroughly, and plated on 0.8% agar plates with 0.5× MS salts (Sigma-Aldrich) and 5 mM MES (2-(*N*-morpholino)ethanesulfonic acid), pH 5.5 (referred to as MS-agar plates). In most cases, seed germination is shown for both “Cold” and “No Cold” treatments. “Cold” seeds were cold stratified in the dark for 4 d at 4°C prior to scoring germination at 22°C in the light. Cold stratification was used to obtain synchronous seed germination in an effort to decrease variance in seed hormone measurements by capturing seeds at the same state. “No Cold” indicates germination directly at 22°C in the light. The 0h timepoint was collected immediately after cold stratification in the dark for 4 d at 4°C (**Figure 1**).

### 2.3 Hormone measurements

Hormone measurements were conducted for L*er* wt, L*er GID1b-OE*, *sly1-2*, and *sly1-2 GID1b-OE* imbibed seeds using 6 to 8 replicates of 100 to 200 mg of seed on a dry weight basis as outlined in **Supplementary Figure 3**. More seeds and replicates were required for timepoints with hormone levels close to the detection limit. Sampling was performed on dry seeds (dry), seeds after imbibition in the dark for 4 d at 4°C (0h), and seeds after imbibition in the dark for 4 d at 4°C plus 12 h in the light at 22°C (12h). Due to limited seed availability, not all genotypes were measured at all imbibition and after-ripening timepoints, see **Supplementary Figure 3** for details. For dry seed samples, the mass of sample input was doubled to allow detection of very low GA levels. Dry samples included 3-4 replicates of 200 mg to 400 mg of seed. An initial experiment with 200 mg and 400 mg input of Columbia wild-type (Col wt) dry seeds indicated that only a minor increase in background resulted and did not obscure the locations of the ABA, GA_4_, or GA precursor peaks (**Supplementary Figures 4A, B**). Examples of GA peaks and associated background for L*er* wt, *sly1-2*, and *sly1-2 GID1b-OE* are shown in **Supplementary Figures 4C–E**. Peaks for L*er GID1b-OE* (not shown) were similar to L*er* wt.

All samples were flash frozen in liquid N_2_ and lyophilized for 48 h. ABA, bioactive GA_4_, and GA precursors and catabolites were measured by liquid chromatography-electrospray ionization-tandem mass spectrometry (LC-ESI-MS/MS) with an Agilent 6410 triple quadrupole LC-MS using column purification to optimize for GA_4_ and GA precursor detection as described previously (Varbanova et al., 2007; Plackett et al., 2012) and for ABA as in Kanno et al. (2010). Hormone levels were quantified relative to internal standards for ABA, GA_4,_ GA_12_, GA_15_, GA_24_, GA_9_, GA_51_, and GA_34_ obtained as described previously (**Supplementary Figure 1**; Varbanova et al., 2007; Plackett et al., 2012). The MS/MS parameters including retention times are shown in **Supplementary Table 1**. Statistical significance was determined by two-tailed Student’s *t*-tests performed using the R statistics package (R Core Team, 2010).

The dry and 0h imbibition timepoints were intended to determine ABA and GA changes with after-ripening earlier in imbibition than had previously been measured. The 12h timepoint was intended to allow comparison of wild-type L*er*, L*er GID1b-OE*, and *sly1-2 GID1b-OE* ABA and GA hormone changes with after-ripening to those of the 2 week after-ripened to 19 month (long after-ripened) *sly1-2* from Ariizumi et al. (2013).

### 2.4 Reanalysis of microarray data

Microarray datasets from previous studies were reanalyzed to obtain insight into possible transcriptional regulation of changes observed in hormone levels. Our previous microarray experiments at the same “dry”, “0h”, and “12h” timepoints used in this study allowed comparisons of hormone and transcriptome data in the same lines at the same timepoints (Nelson and Steber, 2017; Nelson et al., 2017). The raw datasets from Carrera et al. (2007) were obtained from NASCarrays (http://affymetrix.arabidopsis.info/link_to_iplant.shtml). All microarray datasets were analyzed using Robust Multi-array Average (RMA) for background correction and normalization, with significance determined by False Discovery Rate (FDR) with α = 0.05 as described in Nelson and Steber (2017) (Benjamini and Hochberg, 1995; Irizarry et al., 2003). For references to differential regulation in A relative to B (AvsB), up in AvsB means up-regulated in A (or down-regulated in B), whereas down in AvsB means down-regulated in A (or up-regulated in B).

## 3 Results

### 3.1 Strategy for examining the seed ABA and GA hormone levels associated with degree of seed dormancy and stages in dormancy loss and germination

**Figure 2:**
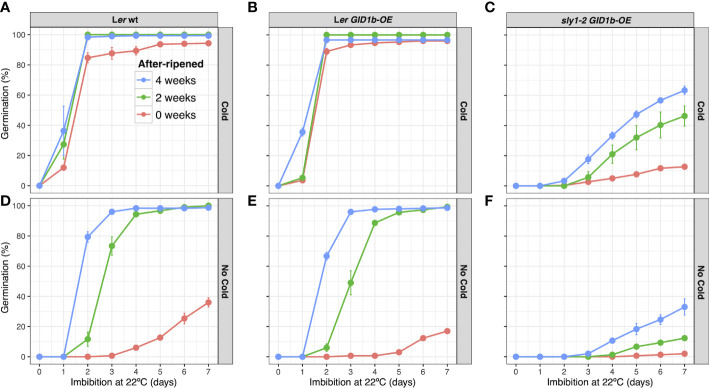
Germination of L*er* wt, L*er GID1b-OE*, and *sly1-2 GID1b-OE* over an after-ripening time course. Percent germination in the light at 22°C of L*er* wt **(A, B)**, L*er GID1b-OE* **(C)**, **(D)**, and *sly1-2 GID1b-OE* **(E, F)**, at 0, 2, and 4 weeks of after-ripening without cold stratification (Cold): **(B, D, F)**, and after cold stratification for 4 d at 4°C in the dark (No Cold): **(A, C, E)**. Error bars represent SE.

Dormancy loss and the germination process are complex multi-dimensional processes influenced by genotype, dry after-ripening time, cold stratification, and time during imbibition. To examine how ABA and GA hormones may regulate degree of seed dormancy and germination capacity, ABA and GA hormone levels were examined in L*er* wt and in the highly dormant GA-insensitive line *sly1-2* (**Figure 1**). Both after-ripening and *GID1b* overexpression can rescue *sly1-2* dormancy. ABA and GA levels associated with dormancy-loss through dry after-ripening and due to overexpression of the *GID1b* GA receptor were examined to see if they were associated with similar hormonal changes. Hormonal differences associated with dormancy due to overaccumulation of DELLA repressor proteins in the *sly1-2* mutant were examined by comparison to the corresponding wild-type, L*er*. Hormone levels were measured in dry seeds (“dry” timepoint), in seeds harvested immediately after cold stratification for 4 d at 4°C in the dark (0h timepoint), and seeds harvested after 12 h imbibition at 22°C in the light following the cold stratification treatment (12h timepoint) for the genotypes indicated in **Figure 1**. Cold stratification was used to synchronize seeds at timepoints in imbibition as they approach germination *per se*, which reduced seed-to-seed variation in our analyses. Based on transcriptome data, the 0h timepoint corresponds to early Phase II and the 12h timepoint to late Phase II of imbibition (Nelson and Steber, 2017). Seeds were harvested at physiological maturity to capture a dormant state. After-ripening timepoints included: no after-ripening 0 week (0wkAR, 0dAR), 2 weeks (2wkAR), 4 weeks (4wkAR), 5 weeks (5wkAR), and 21 months (Long AR) of dry storage. The imbibition timepoints and some of the after-ripening timepoints corresponded with those used in two previous transcriptome studies, allowing comparison of hormone level differences with changes in the expression of GA biosynthesis and catabolic genes (Nelson and Steber, 2017; Nelson et al., 2017). As shown in those previous studies, the *sly1-2* mutant exhibited little or no germination when freshly harvested, and only a mild increase in germination with 4 weeks dry after-ripening (**Supplementary Figure 5**). Long after-ripening, for 21 months, however, resulted in 82% germination in cold stratified seeds. The *sly1-2* dormancy phenotype is also rescued by *GID1b* overexpression (**Supplementary Figure 5**; **Figure 2E**). Previous work showed little change in the transcriptome with *GID1b-OE* rescue of *sly1-2* germination, suggesting that germination may result from non-transcriptional mechanisms, such as an altered hormone profile.

Freshly harvested wild type L*er* (0wkAR) showed mild seed dormancy that was additively relieved by cold stratification and dry after-ripening (**Figures 2A, B**). Whereas *GID1b-OE* resulted in increased *sly1-2* germination (**Supplementary Figure 5**; **Figures 2E, F**), *GID1b-OE* resulted in decreased germination of wild-type L*er* at 0 wk AR (**Figures 2A–D**; **Supplementary Figure 6**). At 0wkAR, L*er* was dormant reaching 27% germination, whereas L*er GID1b-OE* reached only 17% germination by 7 d of imbibition. This negative effect on germination decreased with 2 and 4 weeks of after-ripening, although the percent germination remained lower than untransformed wild type at multiple imbibition timepoints. Although cold stratification is a dormancy-breaking treatment, cold stratification and after-ripening had additive effects (**Figure 2**). This is consistent with the fact that dormant *sly1-2* cannot germinate following cold stratification, but can germinate with both long after-ripening and cold stratification (**Supplementary Figure 5**; Ariizumi and Steber, 2007; Ariizumi et al., 2013).

### 3.2 Hormone changes associated with wild-type Landsberg *erecta* seed imbibition and dormancy loss through dry after-ripening

**Figure 3:**
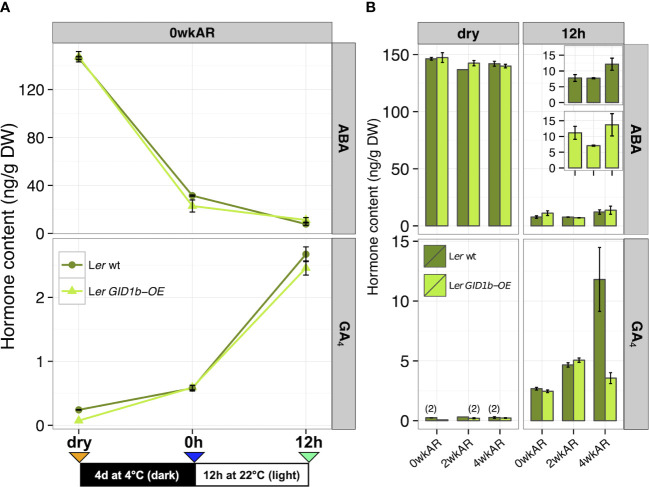
ABA and GA hormone measurements for L*er* (dark green) and L*er GID1b-OE* (light green). **(A)** Measurements across imbibition: dry, 0h, and 12h levels of ABA and GA in dormant 0 week after-ripened (0wkAR) seeds. **(B)** Measurements at a fixed imbibition timepoint (dry or 12h), for comparison of hormone changes with after-ripening for 0, 2, or 4 weeks (0, 2, 4wkAR). Error bars represent SE. “(2)” indicates that endogenous levels were only detected twice, and bars without error bars were only detected once of total replicates assayed.

To examine if there are changes in ABA and GA levels with imbibition of dormant wild-type L*er*, hormone levels were compared with 0wkAR in dry seeds, at 0h, and 12h imbibition timepoints (**Figure 3A**). In dormant seeds, the high ABA levels in dry seeds decreased strongly during cold stratification (dry vs 0h, -114.80 ng/g DW, p = 6.7 x 10^-14^), and also decreased from 0h to 12h of imbibition (-23.73 ng/g DW, p = 1.6 x 10^-10^). GA_4_ levels increased with cold stratification (dry vs 0h, +0.34 ng/g DW, p = 5.9 x 10^-3^), but showed a much stronger increase over Phase II of imbibition (0h vs 12h,+2.09 ng/g DW, p = 9.4 x 10^-10^). Thus, ABA decreases and GA increases with imbibition even in seeds that are not after-ripened. These results are similar to a previous study examining non-dormant L*er* wt seeds imbibed without cold stratification that found high ABA levels in dry seeds that rapidly declined within the first 8 hours of imbibition, and found low GA_4_ levels in dry seeds that did not rise until 24 h of imbibition (Ogawa et al., 2003).

To examine changes in ABA and GA levels with after-ripening, hormone levels were measured at 0, 2, and 4wkAR in both dry and 12h imbibed L*er* seeds (**Figure 3B**). Interestingly, there was no significant decrease in ABA with after-ripening either in the dry seed or the 12h timepoint. In fact, there was a significant increase in ABA content from 2wkAR to 4wkAR in late Phase II (12h) seeds (+4.45 ng/g DW, p = 0.03). No statistically significant changes in GA_4_ levels were detected with after-ripening of dry L*er* wt seeds, due to the fact that GA_4_ levels were close to the detection limit (**Figure 3B**; **Supplementary Figure 7A**; **Supplementary Figure 4C**). In Ler WT At 12h of imbibition, there was a significant increase in GA_4_ hormone levels between 0wkAR and 2wkAR (+1.99 ng/g DW, p = 9.3 x 10^-7^), and between 2wkAR and 4wkAR seeds (+7.15 ng/g DW, p = 9.3 x 10^-3^) (**Figure 3B**). Thus, the increase in germination potential was associated with an increase in GA_4_ levels with early after-ripening, and associated with an increase instead of a decrease in ABA levels from 2wk to 4wk AR at 12h imbibition. Previous work by Ali-Rachedi et al. (2004) in Cvi showed that after-ripened seeds had higher ABA levels than dormant at 12h imbibition, but lower ABA levels than dormant at 24h imbibition, just before seeds germinated. Carrera et al. (2007) made similar observations in ecotype C24. While no measurements was taken at 24h imbibition in the current study, current and previous observations together suggest a model where GA_4_ levels increase with after-ripening at 12h imbibition, before an after-ripening-dependent decrease in ABA levels would likely have been detected at 24h imbibition.

To determine whether the rapid after-ripening of L*er* wt seeds is associated with rapid changes in hormone content, ABA and GA_4_ levels were measured with 1 and 2 days of dry storage at the 12h imbibition timepoint (1dAR and 2dAR in **Supplementary Figure 7B**). The only significant change observed was a slight decrease in GA_4_ levels from 0dAR to 2dAR (-0.41 ng/g DW, p = 0.03) that was not associated with a significant change in percent germination.

### 3.3 The effect of *GID1b-OE* on ABA and GA hormone levels in L*er*

**Figure 4:**
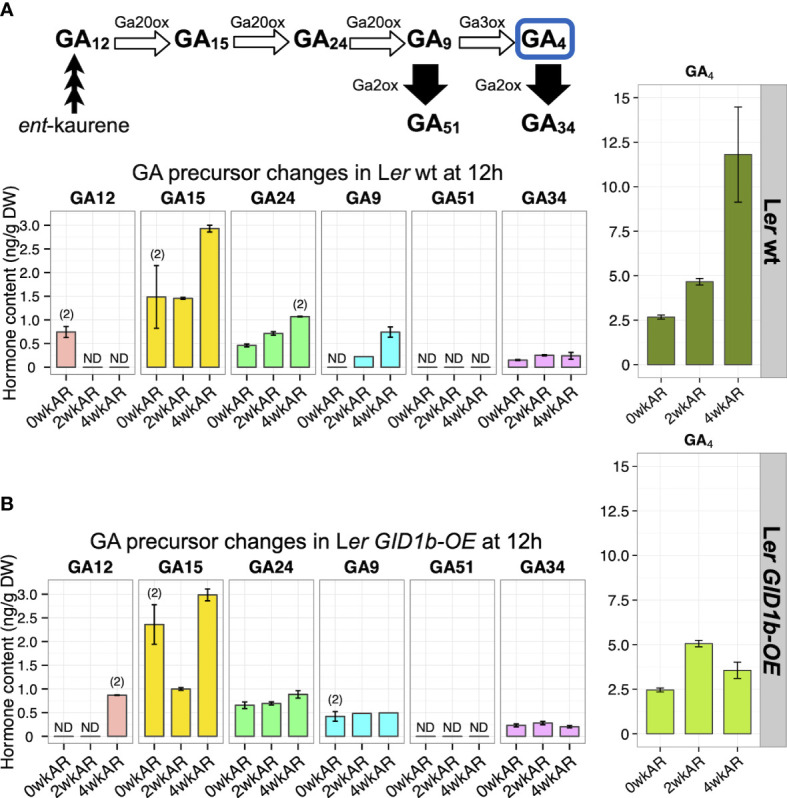
The impact of after-ripening and *GID1b*-over-expression on GA and GA precursor levels in the wild-type background at late Phase II of imbibition. GA precursor levels at 12h of imbibition for **(A)** L*er* wt, and **(B)** L*er GID1b-OE*, at 0, 2, and 4 weeks of after-ripening. Bioactive GA_4_ levels are shown for comparison. For all plots error bars represent SE. “(2)” indicates that endogenous levels were detected twice of 6-7 replicates assayed. Bars without error bars were only detected in one replicate. ND indicates levels were not detectable.

While *GID1b-OE* stimulates *sly1-2* seed germination, it appeared to slow or inhibit the germination of L*er* wt (**Figure 2**; **Supplementary Figure 6**; Ariizumi et al., 2013). To examine whether this reduction in germination potential was associated with altered ABA and GA levels, hormone levels were examined over imbibition and after-ripening (**Figures 3A, B**). In almost every comparison, L*er* wt and L*er GID1b-OE* ABA and GA_4_ hormone levels were indistinguishable. Like L*er* wt, there was no statistically significant decrease in ABA levels with 2wkAR and 4wkAR in L*er GID1b-OE* (p ≥ 0.07). ABA levels appeared slightly higher in the *GID1b-OE* than wt at the 0wkAR 12h timepoint, however this difference was not statistically significant. Whereas L*er* wt GA_4_ hormone levels increased from 0 to 2wkAR and from 2 to 4wkAR, the L*er GID1b-OE* GA_4_ levels increased from 0 to 2wkAR but then *decreased* from 2 to 4wkAR at 12h imbibition (**Figures 3B**, **4B**). This led to lower GA_4_ levels at 4wkAR in the *GID1b-OE* line. However this seems unlikely to influence germination given that both *GID1b* and GA levels are elevated. Thus, the suppressive effect of *GID1b-OE* on wild-type seed germination cannot be well explained by differences in GA or ABA hormone levels.

Next we examined changes in GA precursor and catabolite levels with L*er* wt after-ripening at 12h imbibition (**Figure 4A**). The significant increase in GA_4_ with 2wk and 4wk after-ripening was associated with increases in the precursors GA_15_, GA_24_, and GA_9_. But there was no increase in catabolites GA_51_ and GA_34_. This suggests that increases in GA_4_ are resulting from increasing biosynthesis with little or no increase in turnover. Reanalysis of L*er* wt after-ripened vs dormant transcriptomics data from Carrera et al., 2007 suggests that this pattern may result from significant Phase II increases in the expression of *GA20ox1*, *GA20ox2*, *GA3ox1*, and *GA3ox2*, associated with a small but significant decrease in the levels of the GA turnover enzyme *GA2ox6* (**Supplementary Figure 8**). In Le*r GID1b-OE* at 12h, GA_4_ levels increase at 2wkAR but decrease at 4wkAR (**Figure 3B**). The increase in GA_4_ at 2wkAR is associated only with a decrease in precursor GA_15_ levels (**Figure 4B**). The depletion of GA_15_ and lack of increase in other precursors may explain why there is no sustained increase in GA_4_ levels in the L*er GID1b-OE* line. In dry seeds, there was no significant increase in GA_4_ levels in dry L*er* wt seeds (**Supplementary Figure 7A**), but there was a strong decrease in precursor GA_15_ with L*er* wt after-ripening from 0wk to 2wk and from 2wk to 4wk after-ripening, and a small increase in the penultimate precursor GA_9_ between 0wk and 2 wk after-ripening (**Supplementary Figure 7C**). It is unclear if the increase in GA_9_ is in preparation leads to increased GA_4_ synthesis upon imbibition. In dry L*er GID1b-OE* seeds, there are no statistically significant changes in GA precursors with after-ripening (**Supplementary Figure 7D**).

### 3.4 Differences in ABA and GA levels with after-ripening and imbibition of *sly1-2* and *sly1-2 GID1b-OE* seeds

The *sly1-2* mutant has higher seed dormancy than wild-type L*er* that is reduced either by long after-ripening or by overexpression of GA receptors including *GID1b* (Ariizumi and Steber, 2007; Ariizumi et al., 2013; Nelson et al., 2017). The *sly1-2* mutant showed 0-2% germination and *sly1-2 GID1b-OE* showed 63% germination with cold stratification and 4 weeks of after-ripening at 7 d of imbibition (**Supplementary Figure 5**; **Figures 2E, F**). Since cold stratified *sly1-2* seeds did not germinate well by 4wkAR (8%), hormone measurements were also performed in *sly1-2* seeds following 21 months of dry after-ripening (“Long AR” timepoint, 78% germination). By harvesting *sly1-2 GID1b-OE* at physiological maturity, highly dormant seeds were recovered at 0wkAR (2% germination), and dormancy loss was observed over 2 and 4wkAR (**Figures 2E, F**; **Supplementary Figure 5**). After-ripening and cold stratification acted additively to increase *sly1-2 GID1b-OE* germination potential, reaching 13% at 0wkAR, 48% 2wkAR, and 63% 4wkAR germination at 7 d imbibition. In order to better understand *sly1-2* seed dormancy and dormancy loss, hormone measurements were performed over more detailed after-ripening and imbibition time courses.

**Figure 5:**
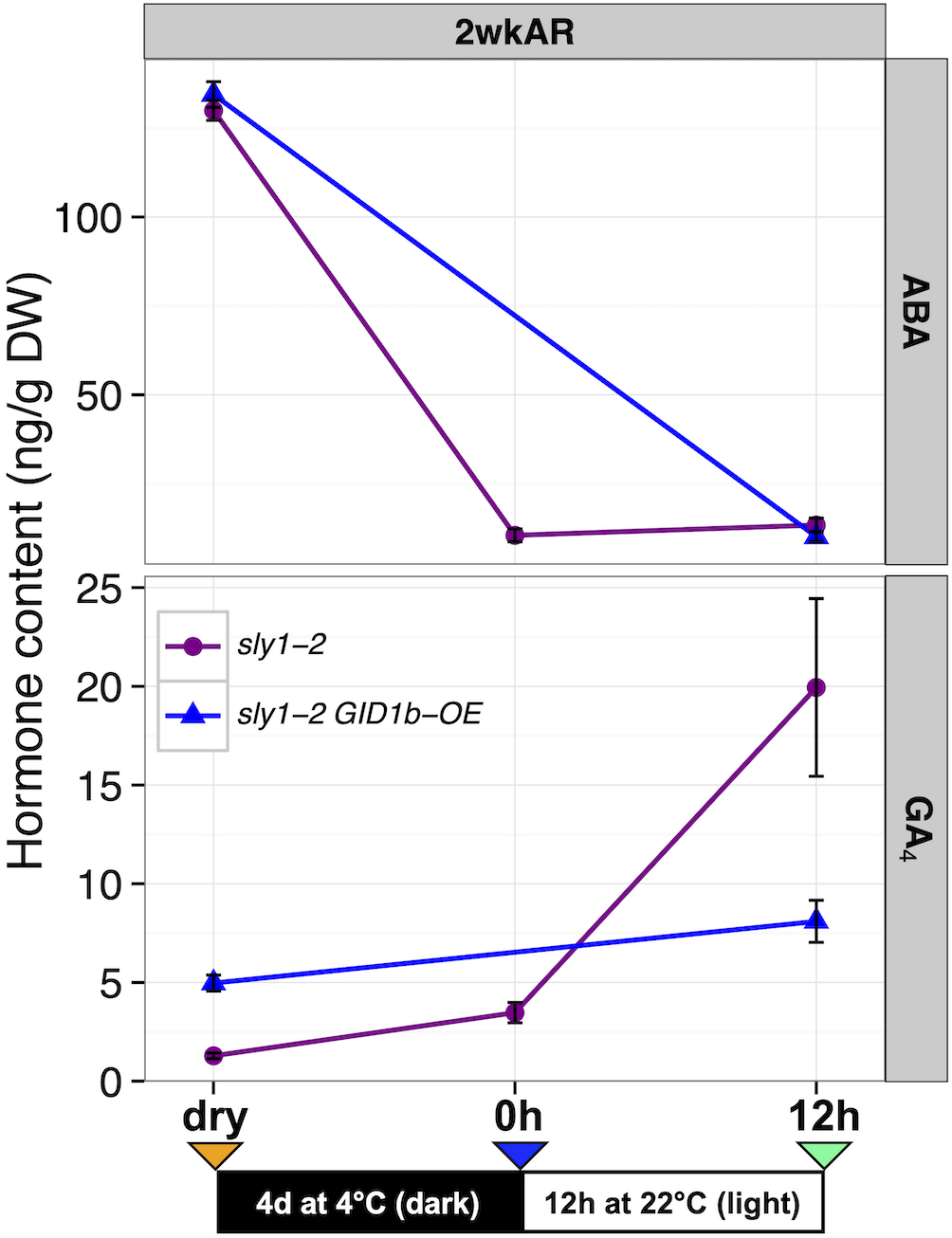
Changes in ABA and GA hormone levels during imbibition in *sly1-2* background lines. Comparison of *sly1-2* (purple) and *sly1-2 GID1b-OE* (blue) ABA and GA hormone levels at the dry, 0h, and 12h imbibition timepoints in 2 week after-ripened (2wkAR) seeds. *sly1-2 GID1b-OE* was not measured at the 0h timepoint. Data for *sly1-2* at 12h was reported in Ariizumi et al., 2013. Error bars represent SE.

To determine whether rescue of *sly1-2* seed germination by *GID1b-OE* (48% versus 0% germination in *sly1-2* after 7d imbibition) is associated with decreased ABA and increased GA_4_ levels, hormone levels were measured over an imbibition time course at 2wkAR (**Figures 2E, F**; **Supplementary Figure 5**). For this experiment, *sly1-2* hormone measurements at dry and 0h timepoints were compared to the previously published 12h imbibition timepoint (**Figure 5**; Ariizumi et al., 2013). The *sly1-2 GID1b-OE* hormone levels were measured in dry seeds and at 12h, but not at the 0h timepoint. ABA levels were very similar in *sly1-2* and *sly1-2 GID1b-OE*, and decreased 117-124 ng/g DW from dry seed to 12h of imbibition. Thus the higher germination potential in *sly1-2 GID1b-OE* cannot be explained by a decrease in ABA accumulation. GA_4_ levels were higher in dry seeds of *sly1-2 GID1b-OE* than in *sly1-2*, but only *sly1-2* GA levels increased significantly with 12h of imbibition. Thus, in spite of the fact that *sly1-2 GID1b-OE* has higher germination potential at 2 weeks of after-ripening than *sly1-2*, its GA_4_ levels do not increase with cold stratification and 12h imbibition. This suggests that *GID1b* overexpression rescues *sly1-2* germination by a mechanism other than decreasing ABA, possibly due to the higher starting GA_4_ content in dry seeds.

**Figure 6:**
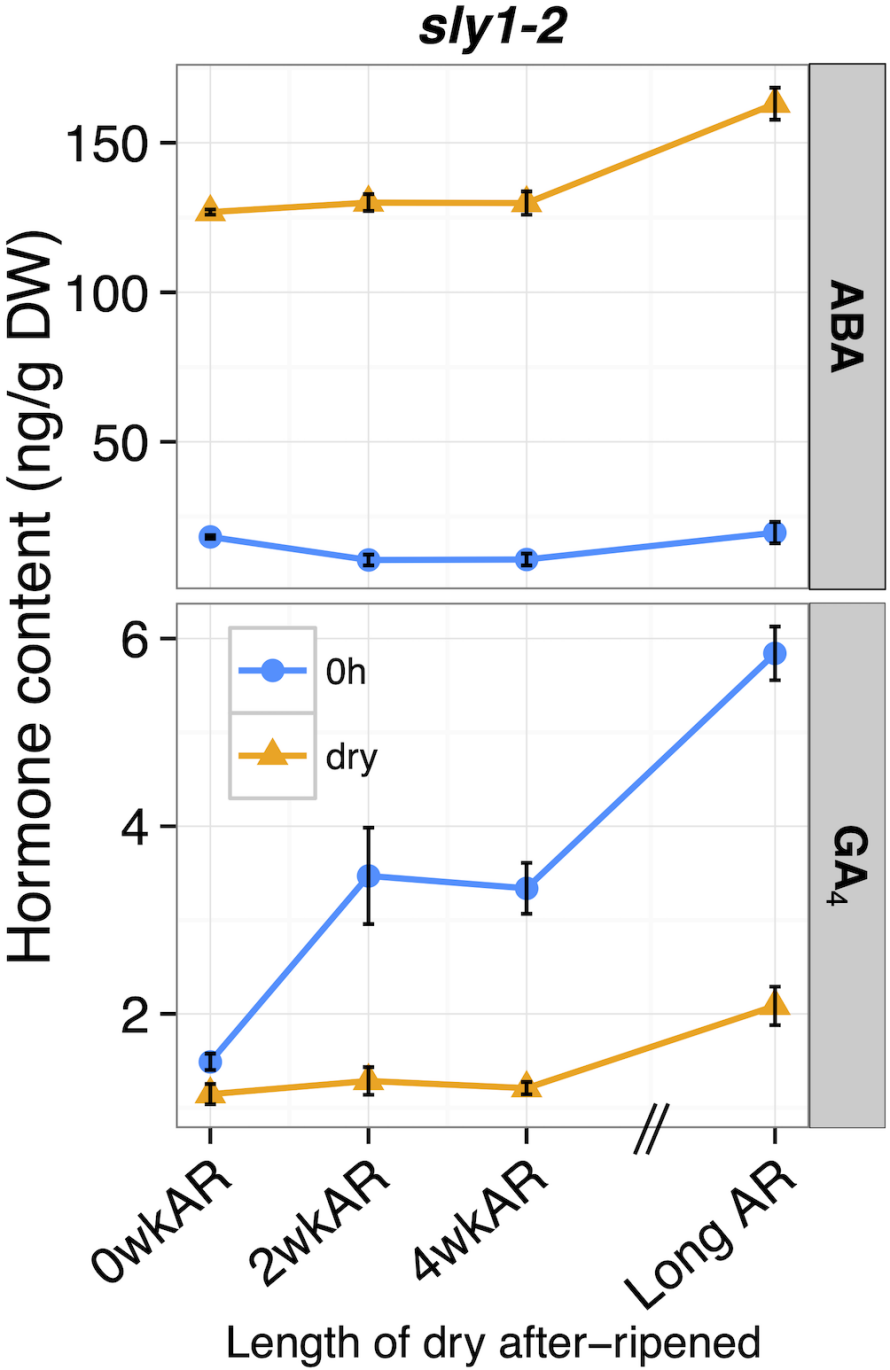
The effect of cold stratification on hormone levels across an after-ripening timecourse in *sly1-2* seeds. ABA and GA_4_ levels of *sly1-2* seeds at dry (orange) and 0h imbibed (light blue) timepoints across after-ripening for 0, 2, 4 weeks (0, 2, 4wkAR), and 21 months (Long AR) of after-ripening. 0h seeds were sampled after cold stratification for 4 d at 4°C in the dark. Error bars represent SE.

While after-ripening caused gradual increases in dry *sly1-2* seed ABA hormone levels over time, there were tremendous changes upon cold imbibition (**Figure 6**). When *sly1-2* ABA levels across an after-ripening time course were compared between dry and cold stratified (dry vs 0h) seeds, cold stratification was associated with clear decreases in ABA levels at all after-ripening timepoints (from -108.61 to -143.43 ng/g DW). Looking at the 0h timepoint, there was an initial significant decrease in ABA levels from 0 to 2wkAR (p = 1.2 x 10^-3^), but no significant change from 2 to 4wkAR. Interestingly, ABA levels increased with long after-ripening (21 months) of *sly1-2* both in dry and in 0h imbibed seeds (2wkAR vs Long AR, dry: p = 0.03, 0h: p = 5.5 x 10^-3^). Thus, while comparison of 2 week to 19 month after-ripened *sly1-2* seed at 12h showed a significant decrease in ABA levels in a previous study, we saw no decrease from 2 weeks to 21 months of after-ripening at the dry or 0h timepoint.

Next we reanalyzed previously published transcriptomic data to examine which ABA biosynthesis and turnover genes are differentially expressed in dormant *sly1-2* seeds versus *sly1-2* seeds whose germination was rescued by long AR or *GID1b* overexpression (Nelson and Steber, 2017; Nelson et al., 2017). ABA levels were higher in dry than in imbibed seeds of all genotypes (**Figures 3**, **5**, **6**). This change was not associated with changes in ABA biosynthesis and turnover gene expression in *sly1-2* seeds (**Supplementary Figure 9**). In fact, ABA biosynthesis genes *ZEP*, *NCED9*, *ABAO*, and *MoCo* were upregulated in 0h and 12h imbibed seeds compared to dry suggesting that active ABA biosynthesis is needed to maintain ABA levels in imbibing seeds. This ABA turnover may result from stored hydrolytic enzyme activity. The increase in ABA levels at 12h imbibition, however, was associated with increased expression of the ABA biosynthesis genes *NCED9*, *ABAO*, and *MoCo*.

After-ripening increased the effectiveness of cold stratification in stimulating GA_4_ accumulation in *sly1-2* seeds (**Figure 6**). In *sly1-2*, the four day cold stratification treatment (0h vs dry timepoint), caused a small increase in GA_4_ levels in seeds without after-ripening at 0wkAR (+0.34 ng/g DW, p = 0.04). However, cold stratification (0h vs dry) of *sly1-2* was associated with significant increases in GA_4_ levels in 2wkAR (+2.19 ng/g DW), 4wkAR (+2.13 ng/g DW), and Long AR (+3.77 ng/g DW) seeds. At the 0h imbibition timepoint, after-ripening was associated with significant increases in GA_4_ levels from 0 to 2wkAR (p = 8.6 x 10^-4^) and from 4wkAR to LongAR (p = 4.2 x 10^-5^). Long after-ripening was associated with increased GA_4_ levels in dry seeds as well. The increasing germination potential of *sly1-2 GID1b-OE* seed with 2wk dry after-ripening was also associated with a strong increase in GA_4_ levels in dry seeds (**Supplementary Figure 10**; **Figure 2F**). However, this was followed by a decrease in GA_4_ at 4wkAR. In *sly1-2 GID1b-OE*, GA_4_ levels increased with 12h imbibition compared to dry seeds with 2wk and 4wkAR. Examination of dry seed GA precursors did not clearly explain why GA_4_ levels increased with after-ripening of dry *sly1-2* and *sly1-2 GID1b-OE* seeds (**Supplementary Figure 11**). There was, however, an increase in GA_12_ with long after-ripening of *sly1-2* at 0h of imbibition (**Supplementary Figure 12**).

**Figure 7:**
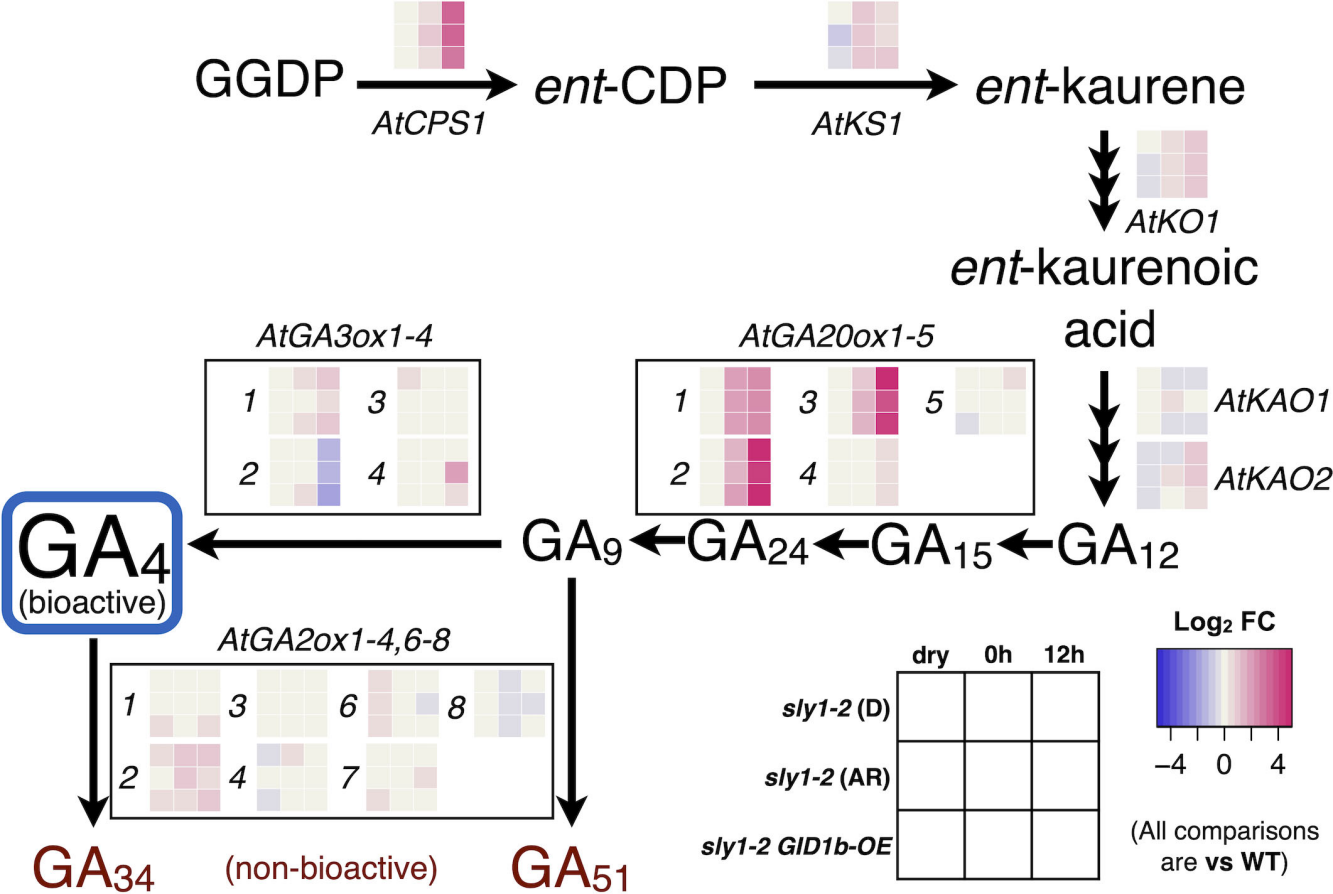
Transcriptional changes in the GA biosynthesis pathway. Transcript levels of GA biosynthesis and catabolic enzymes in dormant (D) 2wk AR *sly1-2*, 19 mo after-ripened (AR, or long AR) *sly1-2*, and in *sly1-2 GID1b-OE* are shown in heat maps relative to levels in L*er* wt (WT) at 2wkAR at dry, 0h, and 12h imbibition timepoints. Magenta indicates a positive and blue indicates a negative fold change (FC) relative to WT. GA_34_ and GA_51_ are products of bioactive GA_4_ turnover. Data come from the reanalysis of microarray datasets from Nelson and Steber (2017) and Nelson et al. (2017). Previous reports did not compare dry and imbibed seed transcript levels.

The transcripts of several GA biosynthesis genes showed increasing expression in *sly1-2* relative to wild-type with imbibition time including *CPS1*, *KS1*, *KO1*, *GA3ox1*, *GA3ox4*, and *GA20ox1*, *2*, and *3* (**Figure 7**). The only strongly upregulated GA biosynthetic transcript with *sly1-2* after-ripening was *GA3ox4* at 12h inhibition. *GA3ox4* is upregulated, but less strongly, with *GID1b-OE*. This is consistent with the increase in GA_4_ levels with *sly1-2* after-ripening at the 12h timepoint, but does not provide an explanation for increase in GA_4_ with *sly1-2* after-ripening at the 0h timepoint (**Supplementary Figure 8**). At the 0h timepoint, GA_4_ levels increased rapidly from 0 to 2wkAR, but were similar from 2 to 4wkAR. By 21 months of after-ripening a significant increase in GA_4_ levels could be observed, suggesting that after-ripening may cause an initial strong increase followed by a gradual increase in GA_4_ levels. Investigation of GA precursors did not provide a clear indication of the mechanisms leading to increasing GA_4_ levels with after-ripening at 0h (**Supplementary Figure 12**).

**Figure 8:**
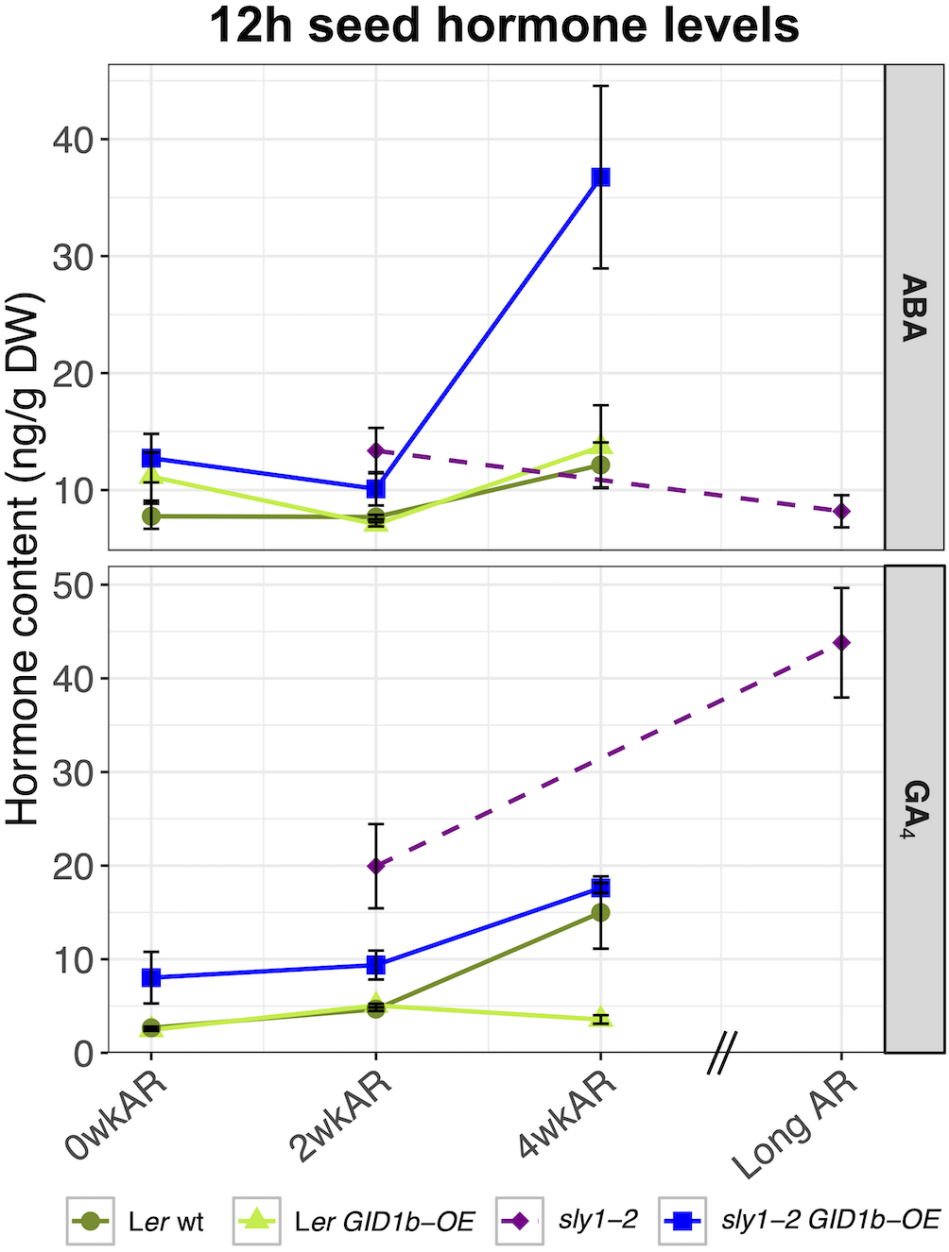
Comparison of ABA and GA changes with after-ripening at 12h of imbibition. L*er* wt (dark green), L*er GID1b-OE* (light green), and *sly1-2 GID1b-OE* (blue) were collected at 0wk, 2wk, and 4wk of after-ripening. The *sly1-2* (purple) measurements at 2wk and 19 months after-ripening from Ariizumi et al. (2013) are plotted for side-by-side comparison with other genotypes. Error bars represent SE.

Next we examined whether the increasing germination potential of *sly1-2 GID1b-OE* with after-ripening was associated with either decreasing ABA or increasing GA_4_ levels at the 12h imbibition timepoint (**Figures 2E**, **8**). The non-significant downward trend in ABA levels from 0 to 2wkAR was followed by a large significant increase in ABA levels from 2 to 4wkAR (+19.15 ng/g DW, p = 9.6 x 10^-5^). Thus, the increase in *sly1-2 GID1b-OE* seed germination was not associated with a decrease in ABA levels. Increasing germination potential was associated with increasing GA_4_ levels with after-ripening of *sly1-2 GID1b-OE* late in Phase II (12h) of imbibition. There was a significant increase in GA_4_ levels from 0 to 2wkAR (4.41 ng/g DW, p = 9.1 x 10^-3^), followed by a larger increase from 2 to 4wkAR (9.51 ng/g DW, p = 1.5 x 10^-5^). The effect of after-ripening on GA precursors and catabolites was also examined in *sly1-2 GID1b-OE* at 12h of imbibition (**Supplementary Figure 13**). The precursor GA_9_ showed increasing accumulation with after-ripening, similar to GA_4_. The GA_9_ catabolite, GA_51_, also increased with 4wkAR. The precursors GA_15_ and GA_24_ levels were highest at 0wkAR, and decreased with after-ripening as GA_9_ and GA_4_ levels increased.

### 3.5 Associations between hormone levels and changes in transcript levels

The *sly1-2* mutant background allows us to examine changes in hormone levels during after-ripening when DELLA protein levels are elevated (Ariizumi and Steber, 2007; Ariizumi et al., 2013). Reanalysis of previously published transcriptomics data enabled us to examine whether changes in hormone levels were associated with the expression of DELLA-regulated genes previously identified in Zentella et al. (2007). The GA 20-oxidase genes were the most prominently *sly1*-upregulated in all three comparisons, dormant *sly1-2* vs L*er* WT (DvsWT), long after-ripened *sly1-2* vs L*er* WT (ARvsWT), and *sly1-2 GID1b-OE* vs L*er* WT (GIDvsWT), at both 0h and 12h timepoints, suggesting that GA 20-oxidase activity is responsible for the high GA levels in *sly1-2* background lines (**Supplementary Figure 14**; Nelson and Steber, 2017). While all of the DELLA-induced regulatory transcripts showed increasing expression with imbibition time, few showed differences in expression in dormant versus after-ripened *sly1-2* (**Supplementary Figure 15**). The exceptions included increased expression of the *RING* and *XERICO* transcripts and decreased expression of the Calmodulin-binding protein *CaM-BP* with *sly1-2* after-ripening.

### 3.6 Comparisons of ABA and GA levels across all genotypes with dry seed after-ripening

**Figure 9:**
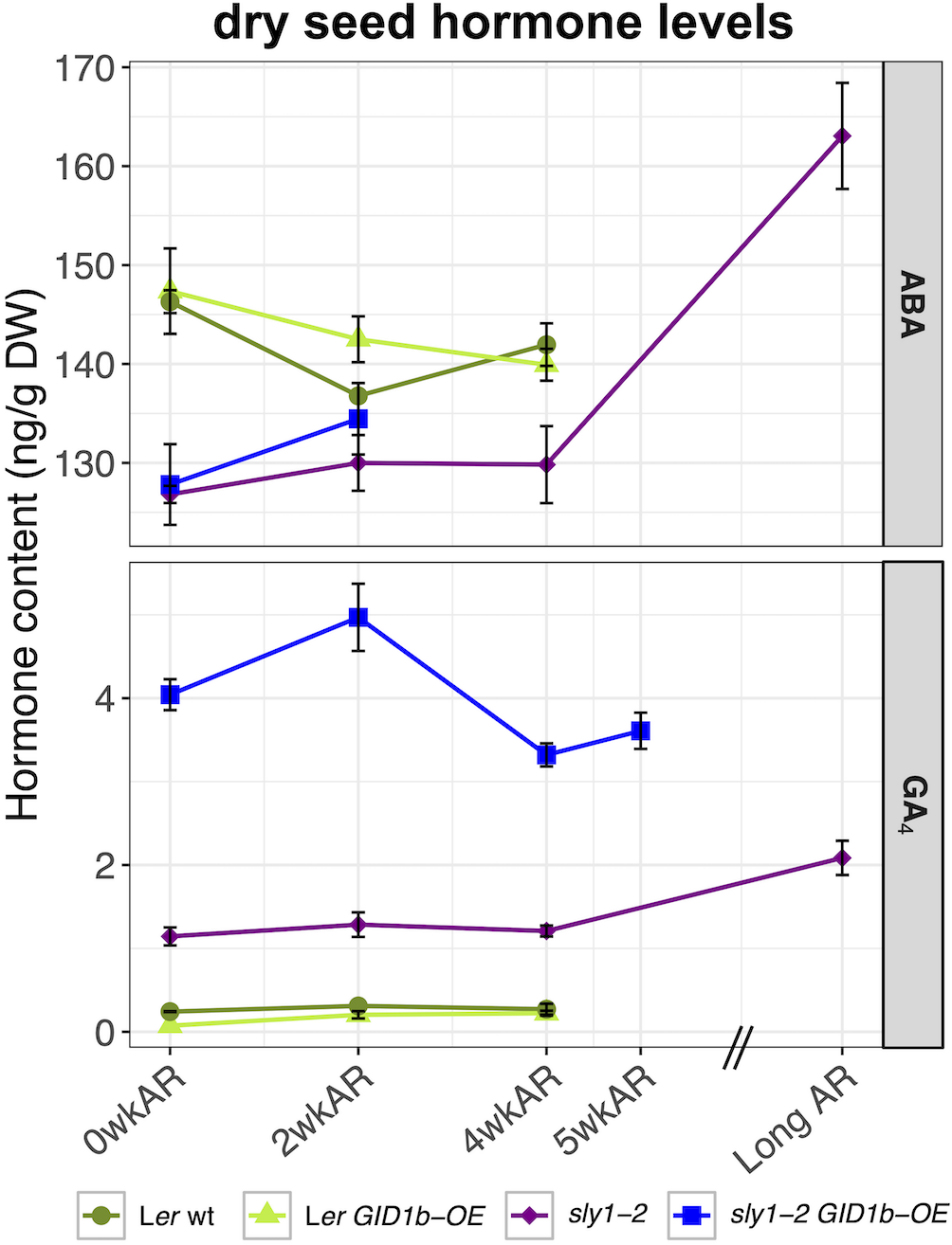
Comparison of dry seed hormone measurements in all genotypes. ABA and GA were measured in L*er* wt (dark green), L*er GID1b-OE* (light green), *sly1-2* (purple), and *sly1-2 GID1b-OE* (blue) across after-ripening timepoints. Error bars represent SE.

To examine the effects of *sly1-2* seed dormancy and of dormancy loss on dry seed ABA and GA levels, hormone levels were compared in all four genotypes over an after-ripening time course (**Figure 9**). If higher ABA levels in dry seeds leads to higher initial seed dormancy, then we would expect higher ABA levels in *sly1-2* seeds. Interestingly, dry seed ABA levels in *sly1-2* and *sly1-2 GID1b-OE* were lower than in L*er* and L*er GID1b-OE* at all measured after-ripening timepoints. The *sly1-2* mutant may have either less ABA biosynthesis or more ABA turnover during seed development (Kanno et al., 2010). ABA did not decline significantly with after-ripening of dry seeds for any of the four genotypes, but did show a downward trend in L*er* and L*er GID1b-OE* from 0 to 4wkAR. Long after-ripening was associated with *increasing* ABA levels in dry *sly1-2* seeds (4wkAR vs Long AR, +33.23 ng/g DW, p = 7.4 x 10^-3^). Thus in dry seeds, the higher seed dormancy of *sly1-2* seeds at 0wkAR was associated with lower ABA levels than wild-type, whereas loss of *sly1-2* seed dormancy was associated with increasing ABA levels. Thus, elevated *sly1-2* dormancy levels do not appear to be the direct result of elevated dry seed ABA levels.

While GA levels were previously known to increase with cold stratification and during seed germination, the effect of dry seed after-ripening was unknown (Yamauchi et al., 2004). Dry seed GA_4_ levels were considerably higher in *sly1-2 GID1b-OE* than in *sly1-2* seeds, and higher in *sly1-2* than in L*er* and L*er GID1b-OE* seeds at 0, 2, and 4wkAR (**Figure 9**). This is consistent with the notion that increased GA_4_ levels result as a feedback effect of a GA-insensitive phenotype. Analysis of *sly1-2* and *sly1-2 GID1b-OE* GA precursors and catabolites did not provide a clear explanation for the highly elevated GA_4_ levels in *sly1-2 GID1b-OE* relative to *sly1-2* and L*er* wt (**Supplementary Figure 11A**). Precursor GA_15_ and catabolite GA_51_ levels were elevated in both *sly1-2* and *sly1-2 GID1b-OE* dry seeds, however the GA_15_ peak was difficult to detect and measure, thus GA_15_ bars are often derived from a single measurement. Long after-ripening (21 mo) of dry *sly1-2* seeds was associated with a significant increase in dry seed GA_4_ levels (+0.88 ng/g DW, p = 1.5 x 10^-2^), as well as with increased levels of the GA precursors GA_12_ and GA_9_, and of the GA catabolites GA_51_ and GA_34_ (**Supplementary Figure 11B**). This suggests that *sly1-2* dry seed after-ripening can result in increased bioactive GA levels through an unknown mechanism. In dry *sly1-2 GID1b-OE*, GA_4_ levels showed a non-significant increase from 0 to 2wkAR, but then decreased significantly from 2 to 4wkAR (p = 0.02), and finally showed no significant increase between 4 and 5wkAR (**Figure 9**).

### 3.7 Comparisons of ABA and GA levels across all genotypes at 12h of imbibition

The effects of after-ripening on ABA and GA levels at 12 h of imbibition were compared in L*er* wt, *Ler GID1b-OE*, *sly1-2*, and in *sly1-2 GID1b-OE* by including *sly1-2* and *sly 1-2 GID1b-OE* data from Ariizumi et al. (2013) (**Figure 8**). ABA levels increased from 2wk to 4wk AR in L*er* wt, L*er GID1b-OE*, and in *sly1-2 GID1b-OE*. The ABA increase was strongest in the *sly1-2 GID1b-OE*. In contrast, ABA levels decreased in long AR versus 2 wkAR in *sly1-2*. We cannot rule out the possibility that longer AR would have a similar effect on the other genotypes. Rescue of *sly1-2* germination by *GID1b-OE* at 2wkAR was associated with a small decrease in ABA compared to *sly1-2*, but no corresponding increase in GA_4_ at 12h. Thus at 12h imbibition, decreasing ABA levels may contribute to rescue of *sly1-2* germination by after-ripening and *GID1b* overexpression. Examination of changes in GA_4_ levels with after-ripening of *sly1-2 GID1b-OE* seeds at 12h showed a significant increase from 2 to 4 weeks of after-ripening (+11.31 ng/g DW, p = 2.92 x 10^-5^) (**Figure 8**). When 12h changes in GA_4_ levels from 2 to 4 weeks of after-ripening for L*er*, L*er GID1b-OE*, and *sly1-2 GID1b-OE* were compared to the previously published changes in 12h GA levels of dormant and long after-ripened *sly1-2*, all lines showed increasing GA_4_ levels with after-ripening, with the exception of the previously discussed decrease in L*er GID1b-OE* GA levels (**Figure 8**). This suggests that an increase in GA_4_ levels by late in Phase II of imbibition is a key part of after-ripening in Arabidopsis. For *sly1-2*, a decrease in ABA levels at 12 hr imbibition but not in dry seeds was associated with increasing germination potential with after-ripening and *GID1b* overexpression.

## 4 Discussion

The antagonism between ABA and GA in the regulation of seed germination is well known, but key questions regarding their relative roles in dormancy loss remain. Previous work found that ABA levels declined at or near the time of seed germination with after-ripening of Arabidopsis, barley, and wheat seeds, apparently due to increasing turnover due to the expression of the ABA catabolic enzyme ABA 8’ hydroxylase (Ali-Rachedi et al., 2004; Millar et al., 2006; Okamoto et al., 2006; Barrero et al., 2009; Liu et al., 2013). In this study we examined the effect of after-ripening on ABA and GA accumulation in dry seeds, and in early and late Phase II of imbibition. This study found that GA_4_ levels increase with after-ripening in dry *sly1-2* seeds or early in imbibition of wild-type seeds, suggesting a role for GA in early dormancy release (**Figures 3**, **6**, **8**, **9**). While decreasing ABA levels were previously associated with germination of after-ripened seeds, we found that elevated ABA levels during Phase II of imbibition in after-ripened seeds, suggesting a role of ABA in preventing premature germination (**Figure 8**). Finally, we learned that dormancy in *sly1-2* is associated with low ABA and elevated GA levels in dry seeds, suggesting that initial *sly1-2* dormancy may result from an ABA-independent mechanism (**Figure 9**).

The fact that after-ripening is correlated with a drop in ABA levels during seed imbibition led to the previous model in which a drop in ABA levels is the primary trigger of seed dormancy loss (Karssen and Laçka, 1986; Ali-Rachedi et al., 2004; Piskurewicz et al., 2008; Barrero et al., 2009). While it was clear that GA is required for Arabidopsis seed germination, the importance of GA in dormancy loss was unclear because bioactive GAs were virtually undetectable until seeds were on the verge of germination (Kanno et al., 2010). The caveat was that researchers could not judge whether bioactive GA levels are undetected because GA is absent or because levels were below the detection threshold. Detection might be challenging because GA synthesis can be restricted to a small number of cells in either shoot or root primordia (Hay et al., 2004). It is possible, for example, that a small increase in bioactive GA accumulation can have a strong effect in seeds poised to respond strongly due to the increased expression of *SLY1* and the GID1 receptors with after-ripening (Lee et al., 2010; Hauvermale et al., 2015). In fact, *GID1b* mRNA and protein levels were shown to increase before ABA hormone levels decreased. Moreover, research in barley showed that after-ripening was associated with increased accumulation of GA precursors before imbibition, suggesting that after-ripening increases the potential to synthesize bioactive GA (Jacobsen et al., 2002). In a previous study, we showed that GA_4_ levels increased and ABA decreased in late Phase II of imbibition with after-ripening of the highly dormant *sly1-2* mutant (Ariizumi et al., 2013). In the current study, we detected increased GA_4_ accumulation associated not with decreased, but with increased ABA content as a result of dry seed after-ripening at 12h imbibition in Ler WT and in *sly1-2 GID1b-OE* (**Figure 3**; **Figure 8**); and in dry seeds of *sly1-2* with long AR (**Figure 6**; **Figure 9**). The only instance where a decrease in ABA levels was seen together with an increase in GA_4_ was at the 12h imbibition timepoint with long AR of *sly1-2* (**Figure 9**; Ariizumi et al., 2013). Previously published work in ecotypes Cvi and C24, showed a decline in ABA levels in after-ripened seeds just as seeds were about to germinate at 24h imbibition (Ali-Rachedi et al., 2004; Carrera et al., 2007). Taken together these observations suggest a model where after-ripening increases germination potential through an increase in GA_4_ levels at 12h imbibition. This is associated with an increase in ABA levels needed to temporarily prevent germination as the seeds complete cellular repair in Phase II at 12h imbibition. ABA levels likely decrease at 24h inhibition enabling normal germination of after-ripened seeds. Future work will need to confirm this model using later imbibition timepoints and possibly longer after-ripening of wild-type seeds.

### 4.1 The relative roles of ABA and GA in controlling dormancy release

ABA is a potent inhibitor of seed germination that establishes seed dormancy during seed maturation and serves as a barrier to germination and embryo growth in mature seeds (Finkelstein et al., 2008; Lee et al., 2010). Based on this, we expected ABA levels to be negatively correlated with germination capacity at each after-ripening timepoint. In contrast, dry seed ABA levels increased as the germination capacity of *sly1-2* seeds increased with long after-ripening for 21 months (**Figure 6**). While *sly1-2* ABA levels decreased with after-ripening at 12h, ABA levels increased from 2 wk to 4 wk of after-ripening in L*er* wt, L*er GID1-OE*, and *sly1-2 GID1b-OE* (**Figure 8**). A similar increase in dry seed ABA content was observed with after-ripening of highly dormant Arabidopsis ecotype Cape Verde Islands (Cvi) at around 6hr of imbibition, followed by a decrease at 12h of imbibition (Ali-Rachedi et al., 2004). Thus, there are other genotypes where increased ABA levels were associated with *increasing* germination capacity prior to germination *per se*.

Previous studies did not observe a decrease in ABA content with after-ripening until seeds were closer to germination at 12-24 hr of imbibition in Arabidopsis ecotypes Cvi and C24 (Ali-Rachedi et al., 2004; Lee et al., 2010). Thus, it appears that ABA levels do not decrease in after-ripened seeds until the seeds are on the verge of germination. Many critical processes must be completed in Phase II of imbibition before a seed can safely germinate, including DNA repair, membrane repair, mitochondrial repair, the initiation of transcription and translation, and the initiation of stored reserve mobilization (reviewed in Bewley et al., 2013). It is possible that ABA serves as a final “checkpoint” to block germination in non-dormant seeds until essential Phase II processes are completed, enabling a successful seed-to-seedling transition. This proposed function is consistent with previous research showing that ABA can serve as a checkpoint blocking the growth, not only of seeds, but of early seedlings when they experience premature drying after germination (Lopez-Molina et al., 2001).

This raises the question of how the seed knows it is ready to complete Phase II and germinate. Future research will need to discover the mechanisms controlling the ABA checkpoint in non-dormant/after-ripened seeds. Previous transcriptional research has implicated the ABA turnover enzyme ABA 8’ hydroxylase (Ali-Rachedi et al., 2004; Millar et al., 2006; Okamoto et al., 2006; Carrera et al., 2007; Barrero et al., 2009; Liu et al., 2013). Research should examine the potential role of ABA 8’ hydroxylase or of ABA glucosyltransferases in lifting ABA repression of the final decision to germinate (Liu et al., 2013; Dong and Hwang, 2014). GA may also be involved in recognizing the safe point for germination. The germination of the *ga1-3* GA biosynthesis mutant can be partly rescued by overexpressing the *GID1* GA receptors (Hauvermale and Steber, 2020). The resulting seedlings die, however, because they are apparently unable to grow and complete development. It is possible *GID1-OE*-rescue of *ga1-3* results in germination before the completion of Phase II repair processes, resulting in death. Future work will need to examine if a similar “ghost” seedling phenotype can be induced by blocking ABA synthesis during Phase II of wildtype germination.

This study provides evidence that GA_4_ may stimulate dormancy loss through after-ripening before ABA levels decline (**Figure 3B**). For *sly1-2*, there is evidence that this increase occurs as early as the dry seed stage and is clearly observable at the 0h timepoint (**Figure 6**; **Figure 9**). Furthermore, in L*er* wt the increase in GA_4_ with after-ripening was observed at 12h of imbibition, when a corresponding decrease in ABA was not yet observed (**Figure 3B**). Thus, it appears that increasing GA_4_ levels may be an early signal triggering dormancy loss through dry after-ripening. ABA levels do not rise during Phase II of dormant seeds, likely because dormant seeds are unable to germinate either because GA_4_ levels are low or for more fundamental reasons, making the ABA checkpoint unnecessary.

The model we propose is that after-ripening results in increasing GA sensitivity as a result of increasing expression of GA signaling proteins such as SLY1 and GID1 (Lee et al., 2010; Hauvermale et al., 2015). This enables seeds to respond to small increases in GA_4_ levels with after-ripening and cold stratification thereby triggering germination-promoting processes. Once this happens, ABA levels increase during early to mid-Phase II to prevent germination until the completion of processes needed to safely undergo the seed to seedling transition, such as DNA repair, restoration of membrane integrity and activation of respiration, transcription and translation. Upon completion of Phade II, ABA levels decline and seeds germinate.

### 4.2 The effects of *GID1b*-overexpression in wild-type L*er* and *sly1-2*

Overexpression of *GID1b* in the *sly1-2* background led to an increase in seed germination, so it was expected that it would have a similar effect in L*er* wt. Interestingly, when germination of L*er GID1b-OE* was compared to that of L*er* wt at three after-ripening timepoints, L*er GID1b-OE* consistently germinated less efficiently than untransformed wild-type (**Figures 2A–D**; **Supplementary Figure 6**). This is consistent with previous observations that *GID1b* can act as a negative regulator of GA signaling. Mutations in *GID1b* can increase stem elongation or increase seed germination in the dark or in under dim lighting suggesting that GID1b negatively regulates GA signaling under some conditions (Griffiths et al., 2006; Ge and Steber, 2018; Hauvermale and Steber, 2020). Future work will need to investigate whether GID1b negatively regulates germination and plant growth *via* DELLA repressors or *via* the recently identified GID1-interacting protein PUX1 (Plant UBX domain containing protein 1) (Hauvermale et al., 2022).

It appears that repression of L*er* seed germination by *GID1b* overexpression does not result from hormonal changes because ABA and GA hormone levels were very similar in L*er* wt and L*er GID1b-OE* (**Figure 3**). The only exception was that from 2 to 4 weeks of after-ripening in late Phase II the GA_4_ hormone levels of L*er* wt increased greatly, while the levels of L*er GID1b-OE* decreased. Examination of L*er GID1b-OE* GA precursor levels in late Phase II were also dissimilar from L*er* wt levels, suggesting that the difference in GA levels is regulated through the biosynthetic pathway (**Supplementary Figures 7C, D**). The GID1b GA receptor is highly sensitive to GA and can bind DELLAs to a certain degree even in the absence of GA (Nakajima et al., 2006; Yamamoto et al., 2010). Thus, one possible model for the decrease in GA_4_ levels with longer after-ripening of L*er* wt is that *GID1b-OE* causes negative feedback regulation of GA biosynthesis. If GA sensitivity increases with after-ripening, and GID1b feedback regulation of GA biosynthesis is a GA signaling response, then once sensitivity reaches a high enough level, elevated GID1b levels lead to a decrease in GA production. In *sly1-2*, where DELLA-proteolysis dependent GA signaling cannot occur, the feedback down-regulation of GA may not be possible. The slightly reduced germination of L*er GID1b-OE* could be due to a decrease in GA levels, although only the 4wkAR GA_4_ levels were lower for L*er GID1b-OE* than L*er* wt at the 12h imbibition timepoint. Further work will need to investigate changes in GA hormone levels at the 0h timepoint or other timepoints earlier in imbibition to determine if *GID1b-OE* inhibits GA accumulation at these timepoints.

### 4.3 High GA levels in *sly1-2* background lines during early and late phase II of imbibition

The *sly1-2* mutation leads to an overaccumulation of GA that is highest in long after-ripened seeds, but still apparent in 2-week-after-ripened dormant *sly1-2* and *sly1-2 GID1b-OE* seeds at late Phase II of imbibition (Ariizumi et al., 2013). It was postulated that the heightened level of GA in *sly1-2* background lines is due to inability to degrade DELLA through the SCF^SLY1^ ubiquitin proteasome pathway. In this model, DELLAs induce GA production as a mechanism of negative self-regulation, but since GA cannot stimulate DELLA-proteolysis in *sly1-2*, both DELLAs and GA accumulate in a feedback loop. The present work investigated bioactive GA_4_ levels in dry and 0h imbibed seeds to determine if *sly1-2* lines had elevated GA levels throughout imbibition. Indeed, GA_4_ levels in all *sly1-2* background lines analyzed in this study were higher than in wild-type in dry, 0h, and in 12h imbibed seeds regardless of after-ripening time (**Figures 3**, **6**, **8**, **9**). We used GA metabolite levels and gene expression data for genes in the GA biosynthesis pathway to determine which steps of the GA biosynthesis pathway and which GA biosynthetic genes are likely responsible for the increased GA accumulation in *sly1-2* background lines (**Figure 7**; **Supplementary Figures 11**-**13**; Nelson and Steber, 2017; Nelson et al., 2017). Transcripts that are likely important points of regulation during after-ripening of *sly1-2* include *KAO1* (*ENT-KAURENOIC ACID OXIDASE1*) and *KAO2*, and *GA3ox4* (**Figure 7**; **Supplementary Figure 8**). Indeed, long after-ripening stimulated an increase in levels of GA_12_, the GA precursor directly downstream of *KAO1* and *KAO2*, in 0h imbibed *sly1-2* seeds (**Supplementary Figure 12**). However, not all changes in ABA and GA_4_ hormone levels observed corresponded to a transcriptional change suggesting that some of the regulation may be posttranscriptional.

If DELLA overaccumulation in *sly1-2* leads to up-regulation of *GA20ox* DELLA targets in imbibed seeds, it is likely that other putative DELLA target genes may show a similar pattern of up-regulation in *sly1-2* vs WT (Nelson et al., 2017). In addition to *GA20ox2*, 17 other putative DELLA targets were defined in Zentella et al. (2007), 10 of which showed a similar pattern of up-regulation to *GA20ox2* in comparisons of *sly1-2* to wild-type L*er* at 0h and 12h of imbibition (**Supplementary Figure 15**). This up-regulation compared to WT was seen even when *sly1-2* germination was rescued by *GID1b-OE* or by after-ripening, suggesting that this increase in expression is due to DELLA overaccumulation. This is consistent with the idea that DELLA overaccumulation in *sly1-2* caused increased GA accumulation through positive transcriptional regulation of DELLA targets in the GA biosynthesis pathway.

Analysis of DELLA-regulated gene expression also helps to understand why ABA levels are high in *sly1-2* mutants. The *XERICO* gene is a DELLA-upregulated positive regulator of ABA biosynthesis (Zentella et al., 2007; Piskurewicz et al., 2008). A previous study found that *XERICO* mRNA levels were slightly down-regulated with after-ripening of *sly1-2*, consistent with a decrease in ABA levels at 12h of imbibition (Ariizumi et al., 2013). Based on transcriptome data, *XERICO* mRNA levels were elevated in *sly1-2* compared to WT at both 0h and 12h of imbibition, even when *sly1-2* germination is rescued by after-ripening and *GID1-OE* (**Supplementary Figure 15**). This is consistent with the observation that ABA levels were higher in imbibing seeds of *sly1-2* lines compared to wild-type L*er* lines at 12h imbibition and 2wkAR(**Figure 8**). In contrast, *sly1-2* ABA levels were lower than WT in dry seeds (**Figure 9**). However, not all changes in ABA hormone levels were associated with the expected changes in *XERICO* expression, again implying that other regulatory mechanisms exist.

### 4.4 The effect of dormancy on induction of GA_4_ during cold stratification

The germination promoting effects of cold stratification are associated with an increase in GA_4_ hormone levels (Derkx et al., 1994). While short periods of cold during imbibition promote germination in a number of Arabidopsis ecotypes, longer periods of cold treatment inhibit germination through the induction of secondary dormancy (Penfield and Springthorpe, 2012). In this study, cold stratification led to increased GA_4_ accumulation in L*er*, L*er GID1b-OE*, and *sly1-2* seeds. Dormant L*er* wt seeds at 0wkAR showed only a small increase in GA_4_ content with cold stratification (**Figure 3A**). A study using long after-ripened L*er* seeds showed GA_4_ levels close to 4 ng/g DW after 4 d at 4°C, much higher than the GA induction that we observed in dormant seeds (Yamauchi et al., 2004). This suggests that dormancy loss through dry after-ripening potentiates the ability of cold stratification to induce GA production. Consistent with this idea, cold stratification at 0wkAR *sly1-2* also showed the least increase in GA_4_ levels (dry to 0h imbibed seed +0.34 ng/g DW, p = 0.04), while long AR *sly1-2* showed the greatest increase in GA_4_ (dry to 0h seeds +3.76 ng/g DW, p = 6.0 x 10^-5^) (**Figure 6**).

### 4.5 Potential mechanisms for dry after-ripening of seeds

Long after-ripening of *sly1-2* was associated with increased ABA and increased GA_4_ levels in dry seeds (**Figure 9**). Because there is little metabolism in a dry seed, it is hard to conclude that increasing GA levels are due to an active biological mechanism. However, they may be functionally relevant upon imbibition. It is possible that similar changes occur in wild-type seeds, but remain undetected due to the fact the wild-type GA levels are much lower than those in *sly1-2*. This observation of altered hormone levels in dry seeds with after-ripening is not without precedent, however. Increased levels of GA precursors were detected with after-ripening of dry barley grain (Jacobsen et al., 2002), and increased ABA levels were detected with after-ripening of dry Cvi and C24 Arabidopsis ecotypes (Ali-Rachedi et al., 2004; Lee et al., 2010). ABA levels were consistently high in dry seeds, and decreased strongly with cold stratification and a further 12h imbibition of *sly1-2* and wild-type L*er*, both with and without *GID1b-OE*. This is consistent with previous work showing that ABA levels declined with cold stratification of L*er* wt following a pulse of far-red light to inhibit germination (Yamauchi et al., 2004).

How can a dry seed acquire the ability to germinate at moisture levels (5-10%) too low from most biological processes? A promising theory is that dry after-ripening leads to the gradual destruction of negative regulators of germination (Bazin et al., 2011; Nelson and Steber, 2017; Bailly, 2019). Gradual damage to negative regulators of germination by oxidation from exposure to air is one way to create a clock allowing time to pass before a dry seed acquires the ability to germinate. Given that the decision to germinate is governed by the balance between ABA and GA hormone signaling, these hormone signaling pathways seem likely targets of such regulation. Bazin et al. (2011) identified fourteen mRNAs that were impacted by oxidation during dry after-ripening. While some of these genes may have been involved in hormone signaling, none encoded hormone biosynthesis or turnover enzymes. However, regulation may not necessarily be at the level of mRNA stability. Potential targets include the hormones themselves, the enzymes involved in hormone biosynthesis or turnover, their transcripts, transcription factors regulating these enzymes, or elements of the signaling pathways.

Key hormone biosynthesis and catabolic enzymes showing regulation by after-ripening are good candidates for such regulation by destruction during after-ripening. Examples of good candidates include genes showing upregulation with *sly1-2* after-ripening including the GA biosynthesis gene *GA3ox4* and the ABA turnover gene *CYP707A3* encoding ABA 8’-hydroxylase (**Figure 7**; **Supplementary Figure 9**). Future work could examine if transcription factors regulating these genes are regulated by oxidation or after-ripening. Reactive oxygen species have been linked to ABA and GA signaling in seeds. For example, ROS have been reported to stimulate GA biosynthesis through transcriptional effects, and H_2_O_2_ in germinating seeds was associated with ABA degradation, possibly through increased ABA catabolism by ABA8’ hydroxylase (Ishibashi et al., 2015; Li et al., 2018; Bailly, 2019; Jurdak et al., 2022).

## Data availability statement

The original contributions presented in the study are included in the article/**Supplementary Material**. Further inquiries can be directed to the corresponding author.

## Author contributions

CS provided the initial research design and obtained funding. SN, MS, YK, and CS designed the experiments and the approach to analysis After-ripening and germination experiments were performed by SN. Hormone measurements were performed in the lab of MS by SN and YK. SN performed hormone extractions and YK performed LCMS runs. SN performed remaining experiments and analyses. CS and SN wrote and edited the article together with YK and MS. All authors contributed to the article and approved the submitted version.
