# Supplementary material for: Seed dormancy loss from dry after-ripening is associated with increasing gibberellin hormone levels in Arabidopsis thaliana
Source: Front Plant Sci. 2023 May 18;14:1145414. doi: 10.3389/fpls.2023.1145414 (PMC10232786; doi:10.3389/fpls.2023.1145414)
Supplement: Supplementary file 1 [file DataSheet_1.pdf]

Supplementary Material

Seed dormancy loss from dry after-ripening is associated with increasing gibberellin hormone levels in *Arabidopsis thaliana*

Sven K. Nelson, Yuri Kanno, Mitsunori Seo, Camille M. Steber*

*** Correspondence:** Camille M. Steber: camille.steber@usda.gov

1. Supplementary Figures

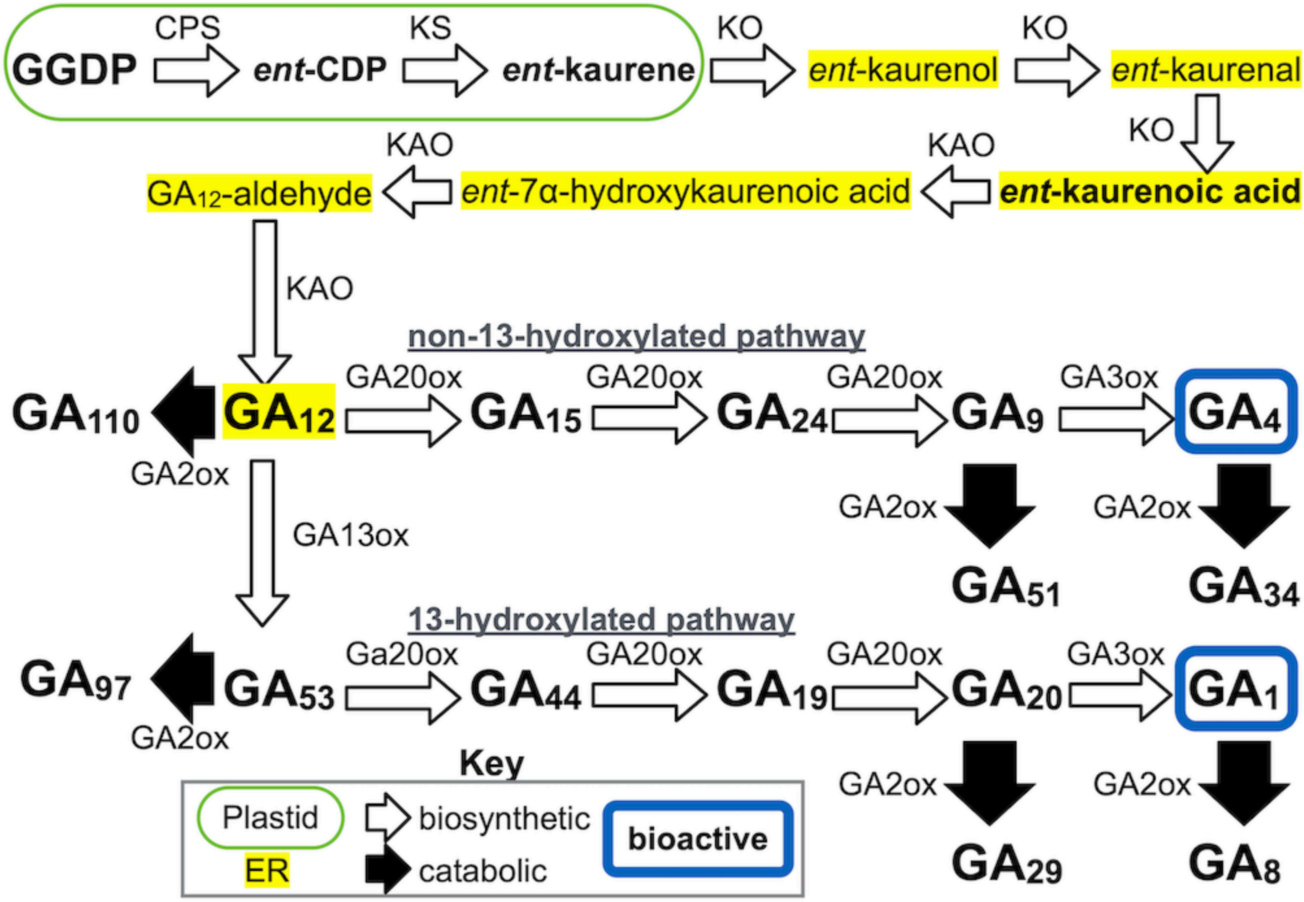

Supplementary Figure 1. GA metabolism. The major bioactive GA in monocots is GA_1, and in dicots, such as Arabidopsis, is GA_4. GA biosynthesis enzymes are: geranylgeranyl diphosphate (GGDP), ent-copalyl diphosphate synthase (CPS), and ent-kaurene synthase (KS) ent-kaurene oxidase (KO), kaurenic acid oxidase (KAO), GA 20-oxidase (GA20ox), and GA 3-oxidase (GA3ox). The GA catabolic enzyme is GA 2-oxidase (GA2ox).

A

B

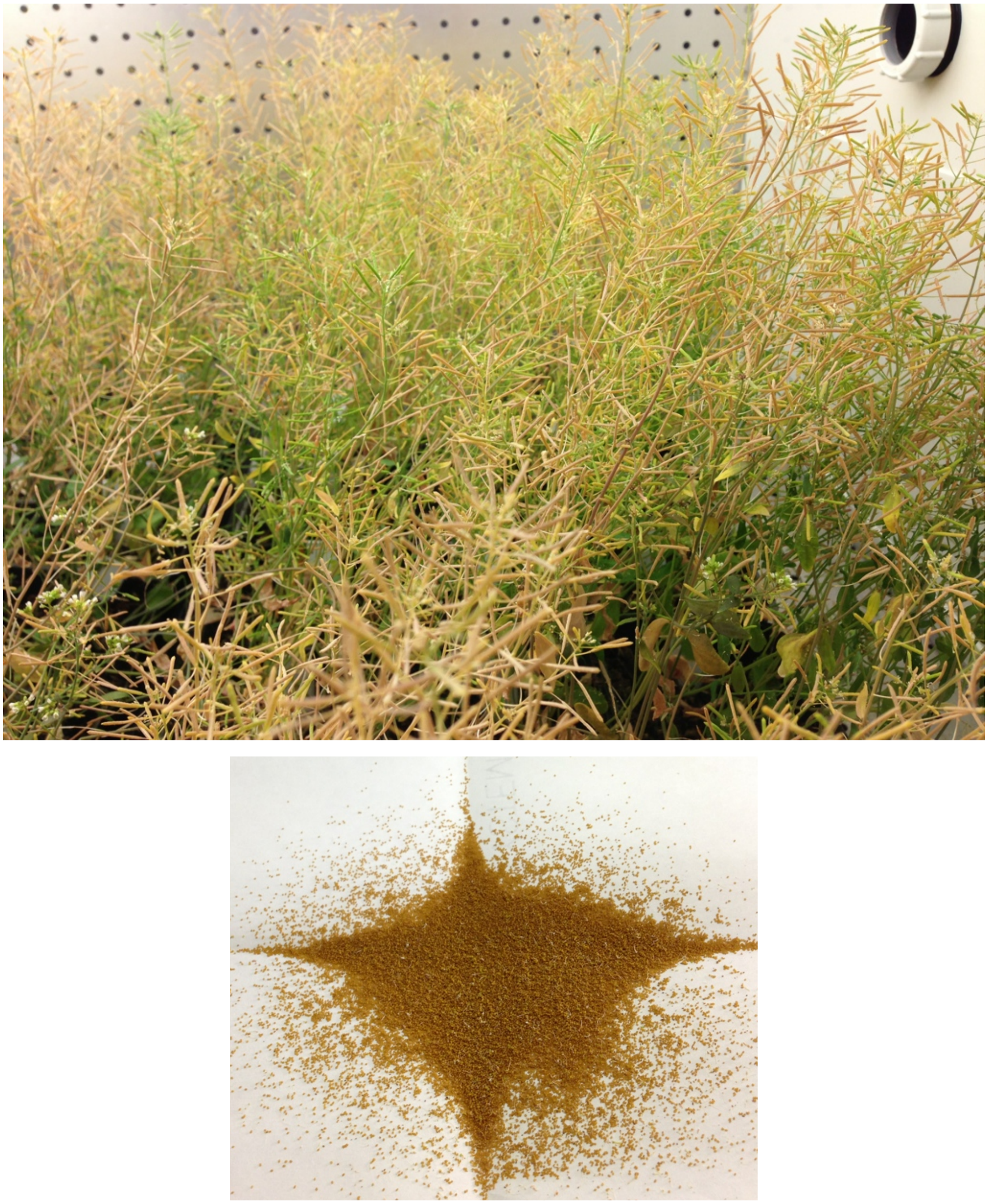

Supplementary Figure 2. Seeds for hormone measurements were harvested at approximately physiological maturity and then stored for the desired after-ripening time. **(A)** Seeds were harvested when plants were approximately half brown and half green. **(B)** Only seeds that freely fell from siliques were collected and put through a fine mesh 3-5 times to remove debris and any “wet” or green seeds. All seeds collected were fully brown as shown.

Line	after-ripening time	imbibition timepoint	reps	measured/detectable		X mg ea
				GA_4	ABA	
wild-type (L*er*)	0wkAR (aka	dry seed	3	2	3	400 mg
		0h imbibed	7	7	7	200 mg
		12h imbibed	7	7	7	200 mg
	1dAR	12h imbibed	7	7	7	200 mg
	2dAR	12h imbibed	7	7	7	200 mg
	2wkAR	dry seed	3	1	1	400 mg
		12h imbibed	7	7	7	200 mg
	4wkAR	dry seed	3	2	3	400 mg
		12h imbibed	6	5	6	200 mg
		**Total samples:**	50			
L*er GID1b-OE*	0wkAR	dry seed	3	1	3	400 mg
		0h imbibed	7	7	7	200 mg
		12h imbibed	8	8	8	200 mg
	2wkAR	dry seed	4	2	4	400 mg
		12h imbibed	8	8	8	200 mg
	4wkAR	dry seed	3	3	3	400 mg
		12h imbibed	7	7	7	200 mg
		**Total samples:**	40			
sly1-2	0wkAR	dry seed	4	4	4	400 mg
		0h imbibed	8	8	8	200 mg
	2wkAR	dry seed	4	4	3	400 mg
		0h imbibed	8	6	8	200 mg
	4wkAR	dry seed	4	3	3	400 mg
		0h imbibed	8	8	8	200 mg
		**Total samples:**	12			
	21 month after-ripened	dry seed	3	3	3	200 mg
		0h imbibed	6	6	6	100 mg
		**Total samples:**	9			
sly1-2 GID1b-OE	0wkAR	dry seed	3	3	3	400 mg
		12h imbibed	6	6	6	200 mg
	2wkAR	dry seed	3	3	3	400 mg
		12h imbibed	6	5	6	200 mg
	4wkAR	dry seed	3	3	0	400 mg
		12h imbibed	6	6	5	200 mg
	5wkAR	dry seed	4	4	0	400 mg
		**Total samples:**	31			
		**Total samples**	142			

Supplementary Figure 3. Strategy for seed time points in after-ripening (AR) (wk is weeks) and in imbibition (h is hours), including the number of replicates (reps), the number of successful measurements for each hormone, and the dry weight in mg of seeds used for hormone measurement.

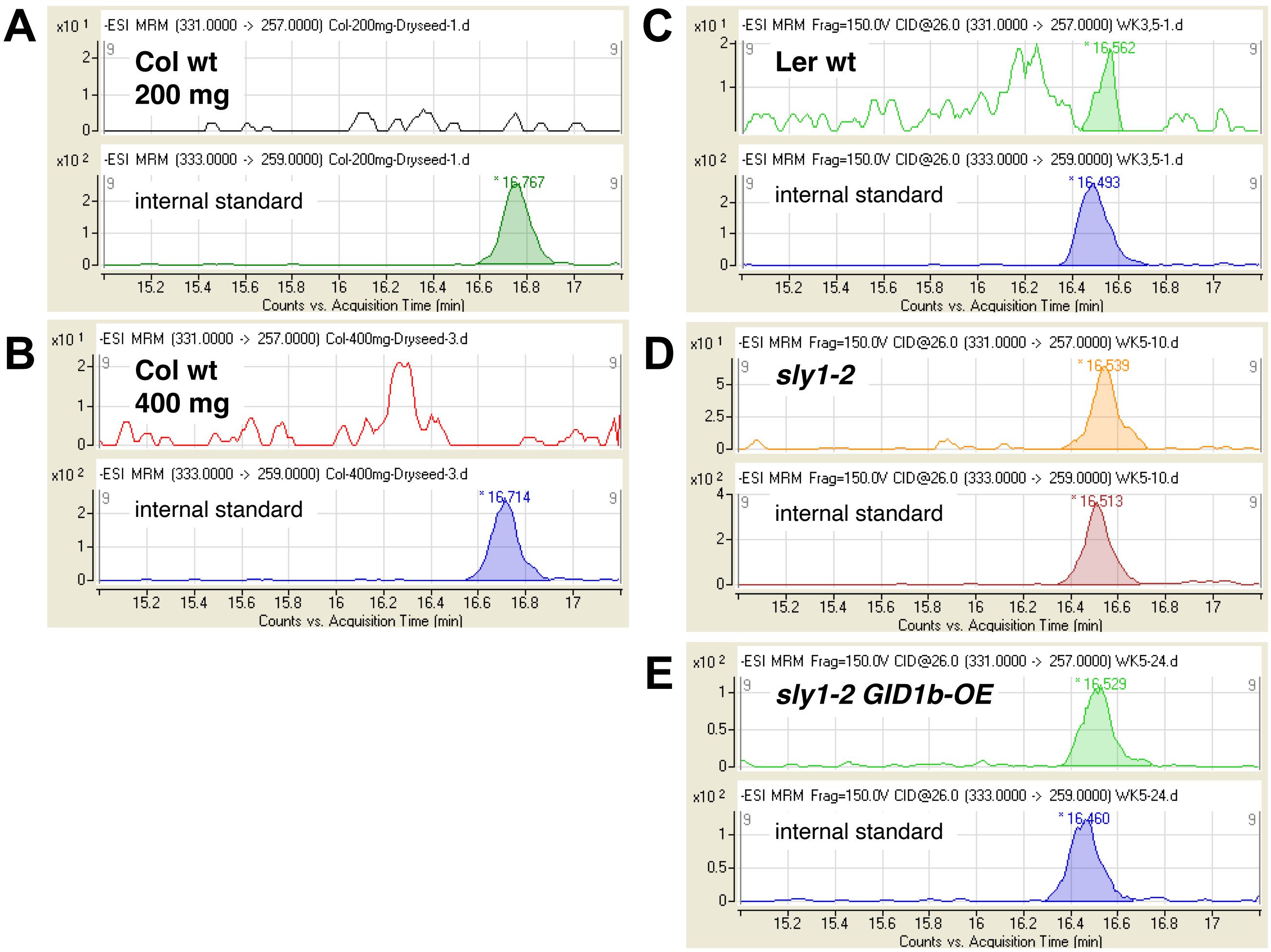

Supplementary Figure 4. Peak calling for hormone data analysis in dry seeds. An initial experiment was performed using **(A)**, 200 mg and **(B)**, 400 mg of dry Col wt seeds to determine if an increase in background would prevent GA detection for doubled samples. For dry seed samples of **(C)**, L*er* wt, **(D)**, *sly1-2*, and **(E)**, *sly1-2 GID1b-OE*, GA could be quantified from 400 mg of seed. L*er* wt measurements were close to the detection threshold, but *sly1-2* and *sly1-2 GID1b-OE* were clearly detectable.

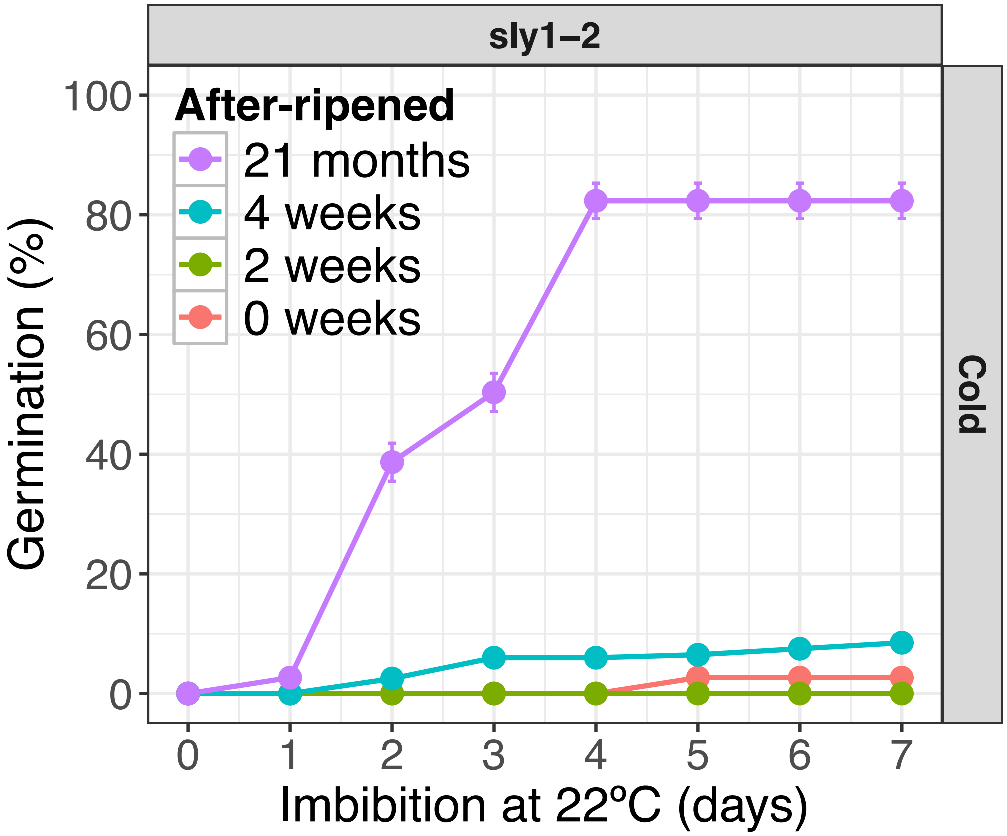

Supplementary Figure 5. Germination of *sly1-2* after-ripened for 0, 2, 4 weeks, and 21 months. It has been previously demonstrated that *sly1-2* is highly dormant at 1 month or less of after-ripening and limited seed quantities were available from the batch of seeds used for hormone measurements so only 21 month after-ripened data is from the same seed batch as the hormone measurements to verify dormancy loss in long after-ripened *sly1-2*. For the 0 week AR timepoint, data shown is from the seed batch used in Nelson et al. (2017) paper, for the 2 week AR timepoint, data shown is from the seed batch used in Nelson and Steber (2017). The 4 week AR timepoint used a separate batch of *sly1-2*. In all cases, storage location and conditions were the same. Seeds were cold stratified in the dark for 4 d at 4°C, then moved to the light at 22°C where germination was scored daily. Error bars represent SE.

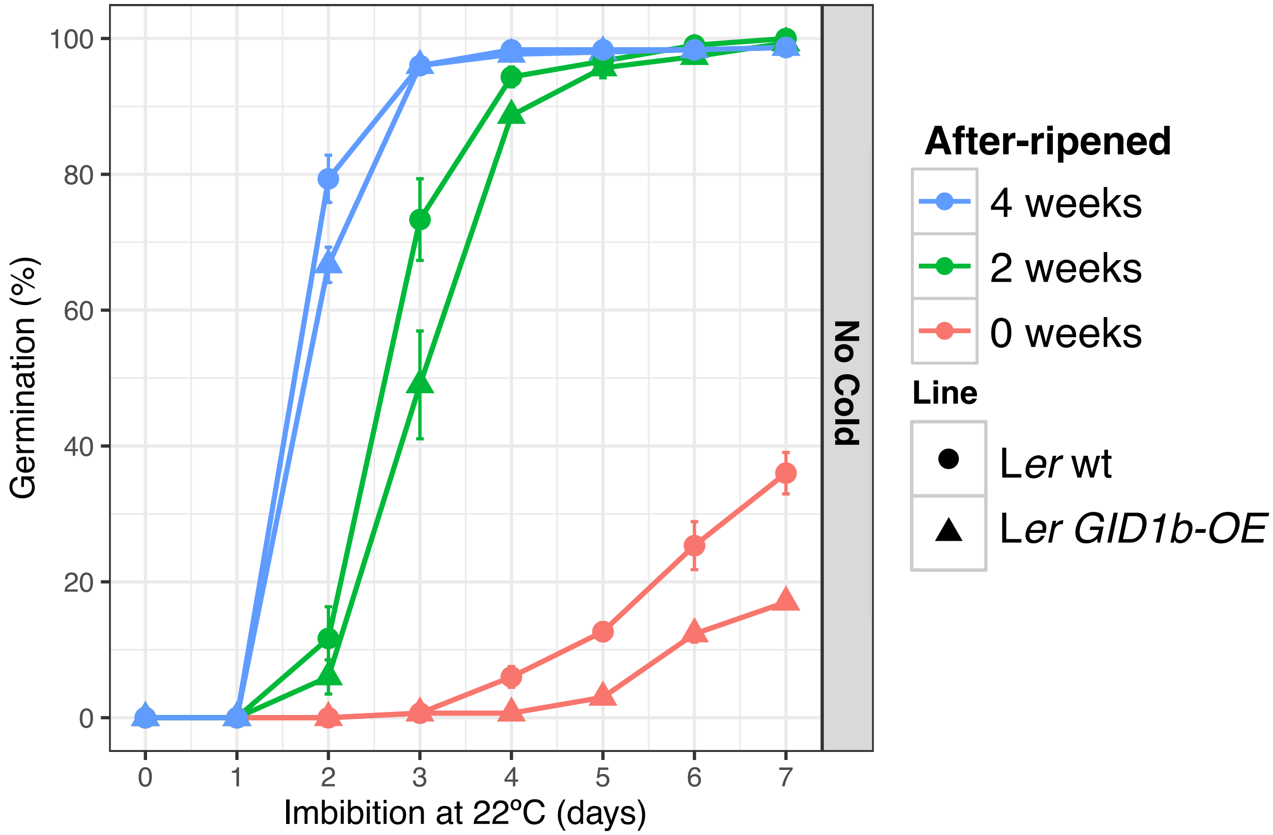

Supplementary Figure 6. Germination of L*er* wt (filled circle) and L*er GID1b-OE* (triangle) at 0, 2, and 4 weeks of after-ripening without cold stratification. Data is the same as in Figure 2B and D, replotted on the same axis for comparison of L*er* wt to L*er GID1b-OE* germination. Error bars represent SE.

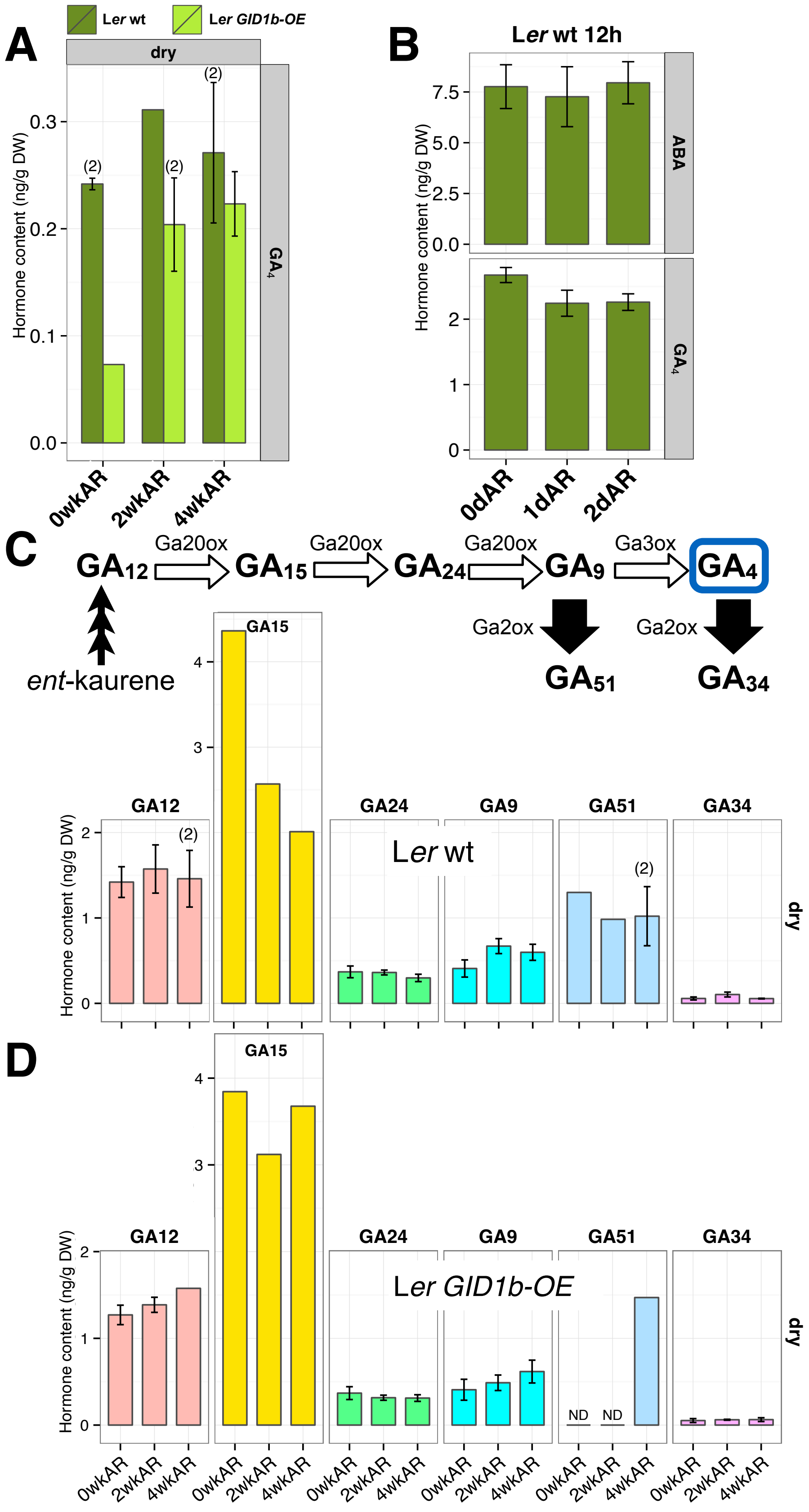

Supplementary Figure 7. L*er* wt and L*er* *GID1b-OE* ABA, GA, and GA precursors. **(A)** Dry seed GA levels for L*er* (dark green) and L*er* *GID1b-OE* (light green) at 0, 2, and 4 weeks of after-ripening

were very low, close to the threshold of detection. **(B)** L*er* wt GA levels at 12h for 0, 1, and 2 day after-ripened seeds. **(C)** Dry seed levels of GA precursor for L*er* wt at 0, 2, and 4 weeks of after-ripening. **(D)** Dry seed levels of GA precursor for L*er* *GID1b-OE* at 0, 2, and 4 weeks of after-ripening. For all plots error bars represent SE. "(2)" indicates that endogenous levels were detected twice of 3-4 replicates **(A, C, D)**, or of 7 replicates **(B)** that were assayed. Bars without error bars were only detected in one replicate. ND indicates the hormone was not detectable.

ID	Gene	Early Phase II[a] logFC	Early Phase II[a] adj.P.Val	Late Phase II[b] logFC	Late Phase II[b] adj.P.Val
**L*er* wt ARvsD[c]**					
At1g05160	KAO1			0.52	1.62×10^{-02}
At2g32440	KAO2			0.58	3.63×10^{-02}
At4g25420	GA20ox1			0.65	9.87×10^{-03}
At5g07200	GA20ox3			1.35	9.29×10^{-03}
At1g15550	GA3ox1			1.44	1.51×10^{-03}
At1g80340	GA3ox2			1.05	7.36×10^{-03}
At1g02400	GA2ox6			-0.45	4.09×10^{-03}
***sly1-2* ARvsD[d]**					
At1g05160	KAO1	0.62	2.97×10^{-02}	—	
At2g32440	KAO2	0.90	2.18×10^{-04}	—	
At5g51810	GA20ox2	—		-1.20	8.36×10^{-07}
At5g07200	GA20ox3	—		-0.80	5.18×10^{-03}
At1g80330	GA3ox4	—		1.88	7.29×10^{-08}

[a] 0h timepoint
[b] 12h timepoint (cold) for sly1-2 or 24 h (no cold) for L*er* wt
[c] L*er* wt (After-ripened) vs L*er* wt (Dormant), 24 h no cold, Carrera et al., 2007
[d] *sly1-2*(After-ripened) vs *sly1-2*(Dormant), Nelson and Steber, 2017

Supplementary Figure 8. Microarray reanalysis to identify GA biosynthesis and catabolic genes that are differentially regulated in dormant (D) and after-ripened (AR) seeds of L*er* and *sly1-2*. *sly1-2* ARvsD dataset from Nelson and Steber (2017), Ler ARvsD dataset from Carrera et al. (2007).

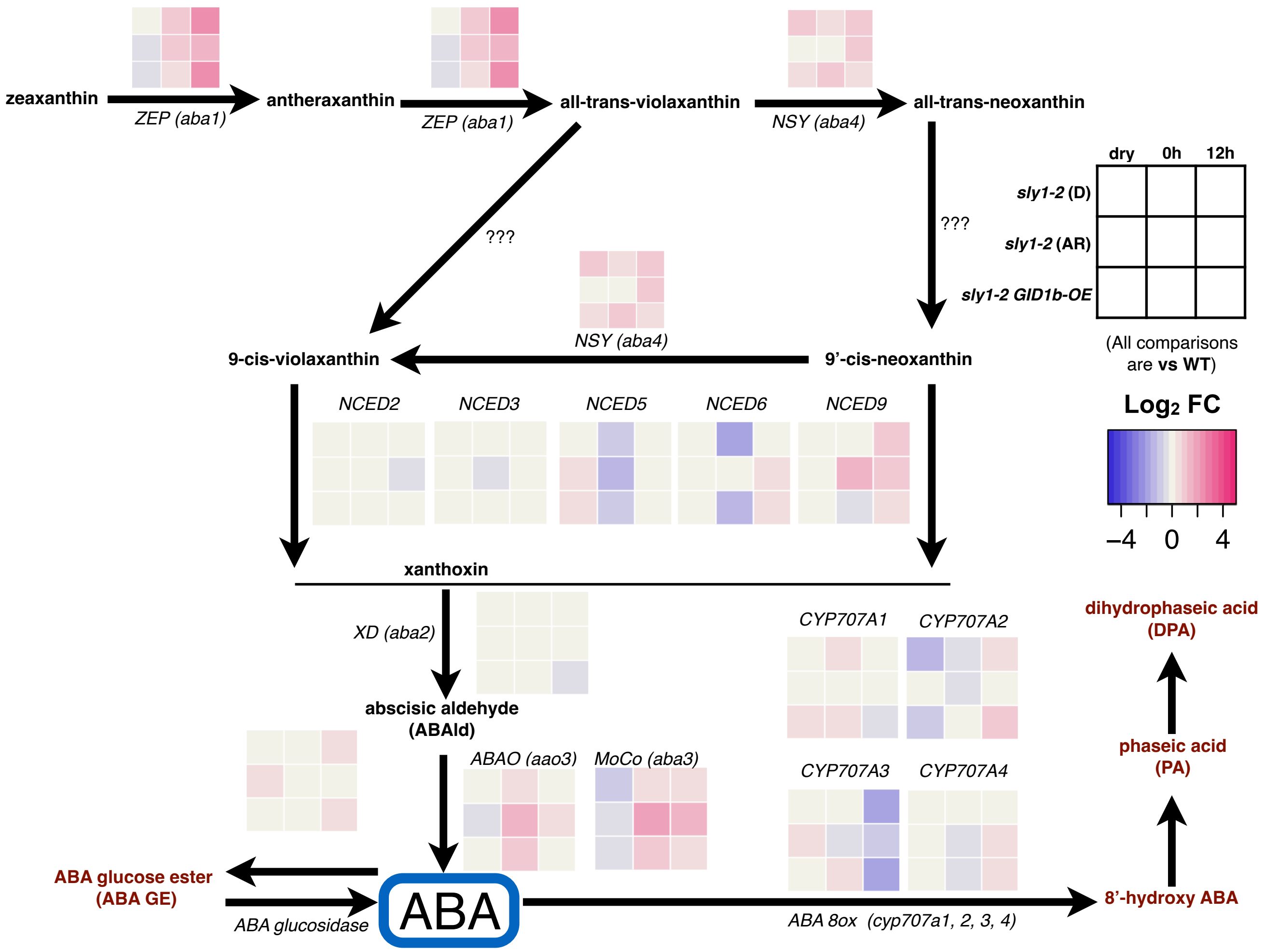

Supplementary Figure 9. Transcript levels of ABA biosynthesis and catabolic enzymes in dormant (D) 2wk AR *sly1-2*, 19 mo after-ripened (AR, or long AR) *sly1-2*, and in *sly1-2 GID1b-OE* are shown in heat maps relative to levels in L*er* wt (WT) at 2 wkAR at dry, 0h, and 12h imbibition timepoints. Magenta indicates a positive and blue indicates a negative fold change (FC) relative to WT. The immediate products of ABA turnover are the inactive ABA glucose ester and 8'hydroxy-ABA. The CYP707A genes are the family of ABA 8'hydroxylases. Data come from the reanalysis of microarray datasets from Nelson and Steber (2017) and Nelson et al. (2017). Previous reports did not compare dry and imbibed seed transcript levels.

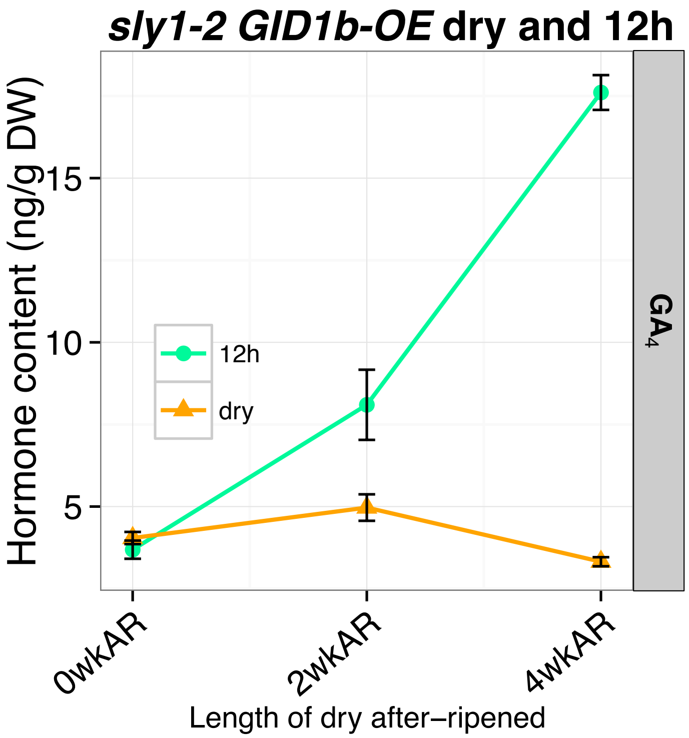

Supplementary Figure 10. Comparison of GA_4 levels of *sly1-2 GID1b-OE* seeds at dry (orange) and 12h imbibed (green) timepoints across after-ripening for 0, 2, and 4 weeks. Error bars represent SE.

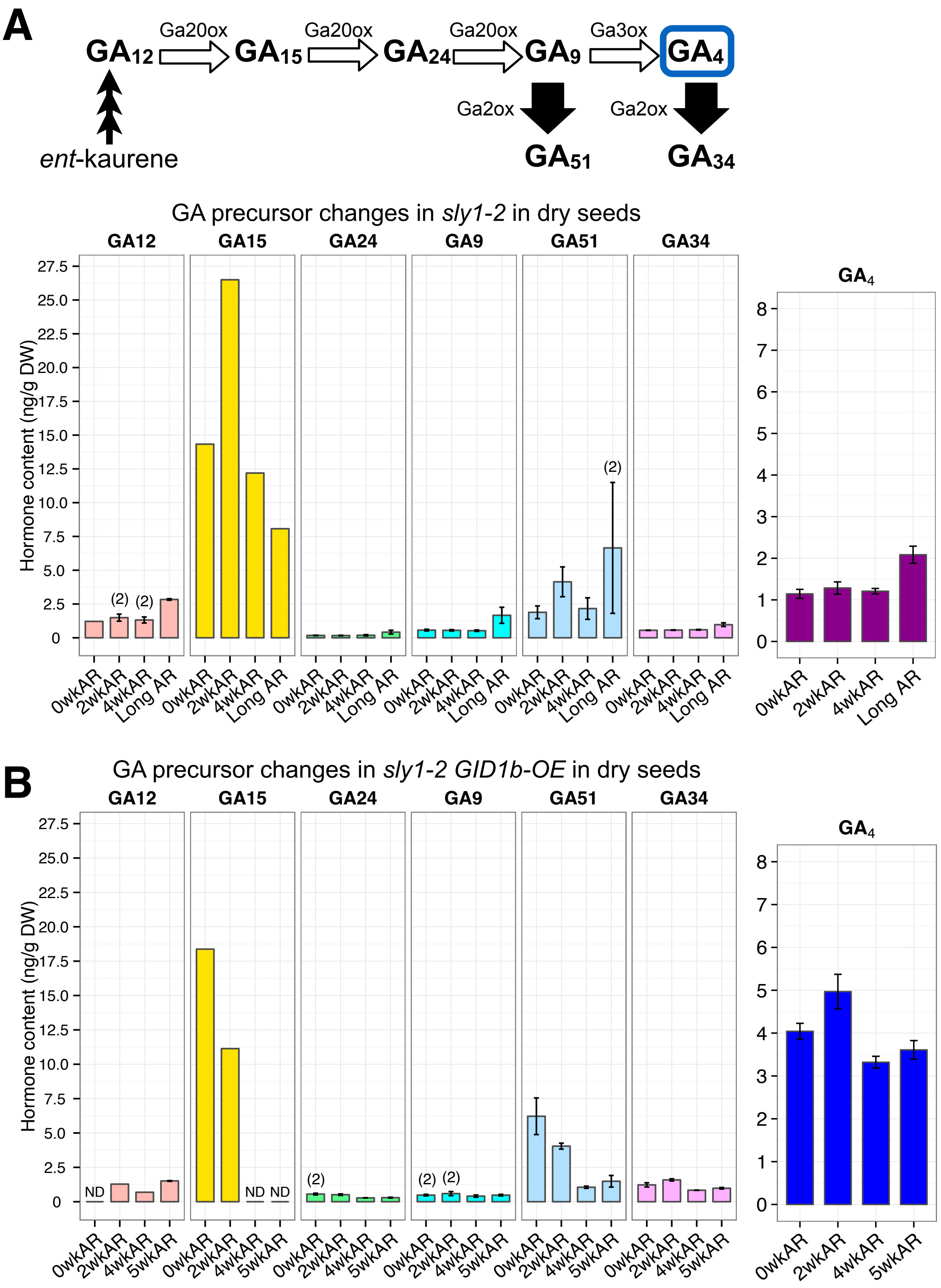

Supplementary Figure 11. GA precursor and catabolite data for dry *sly1-2* and *sly1-2 GID1b-OE* lines. Dry seed GA precursor measurements for **(A)**, *sly1-2* at 0h, and **(B)**, *sly1-2 GID1b-OE* at 12h imibibition at 0, 2, and 4 weeks of after-ripening (0, 2, 4wkAR). Long AR seeds were after-ripened

for 21 months. Bioactive GA_4 levels are shown for comparison on the right. “(2)” indicates that endogenous levels were only detected twice, and bars without error bars were only detected once of total replicates assayed. Error bars represent SE.

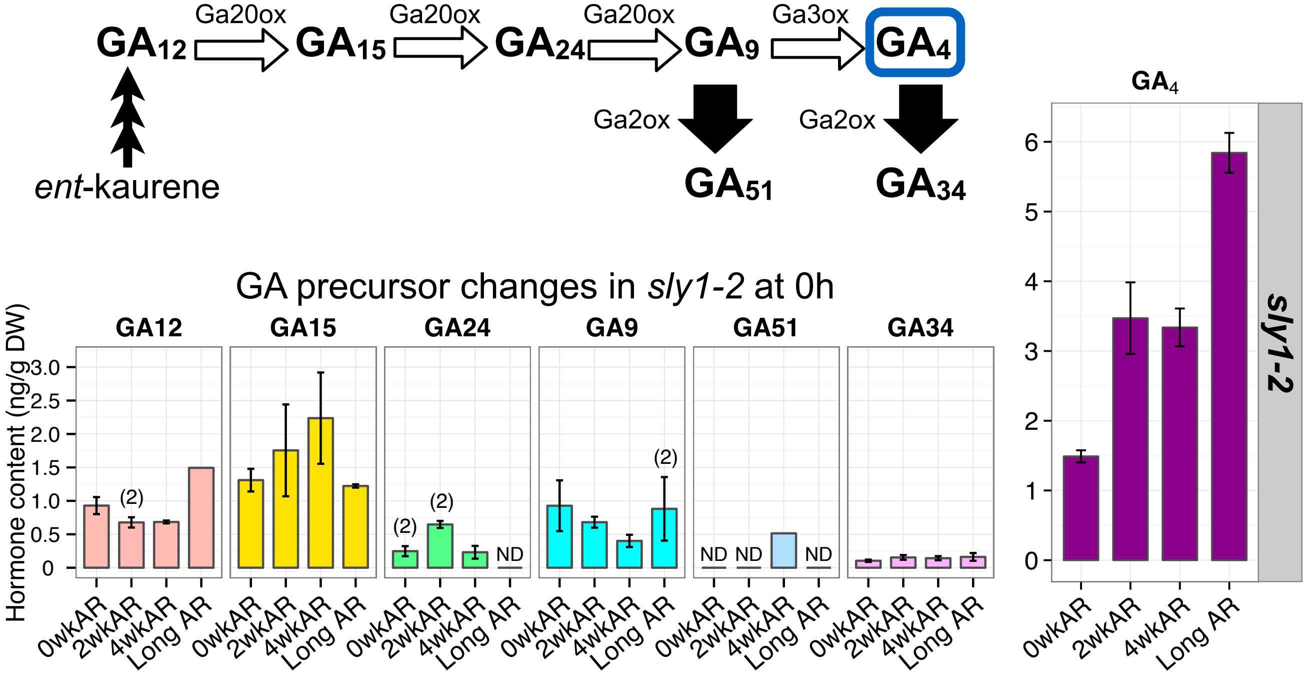

Supplementary Figure 12. GA precursor and catabolite measurements for 0h imbibed *sly1-2* at 0, 2, and 4 weeks of after-ripening (0, 2, 4wk, and long AR). Bioactive GA_4 levels are shown for comparison. "(2)" indicates that endogenous levels were only detected twice, and bars without error bars were only detected once of total replicates assayed. Error bars represent SE.

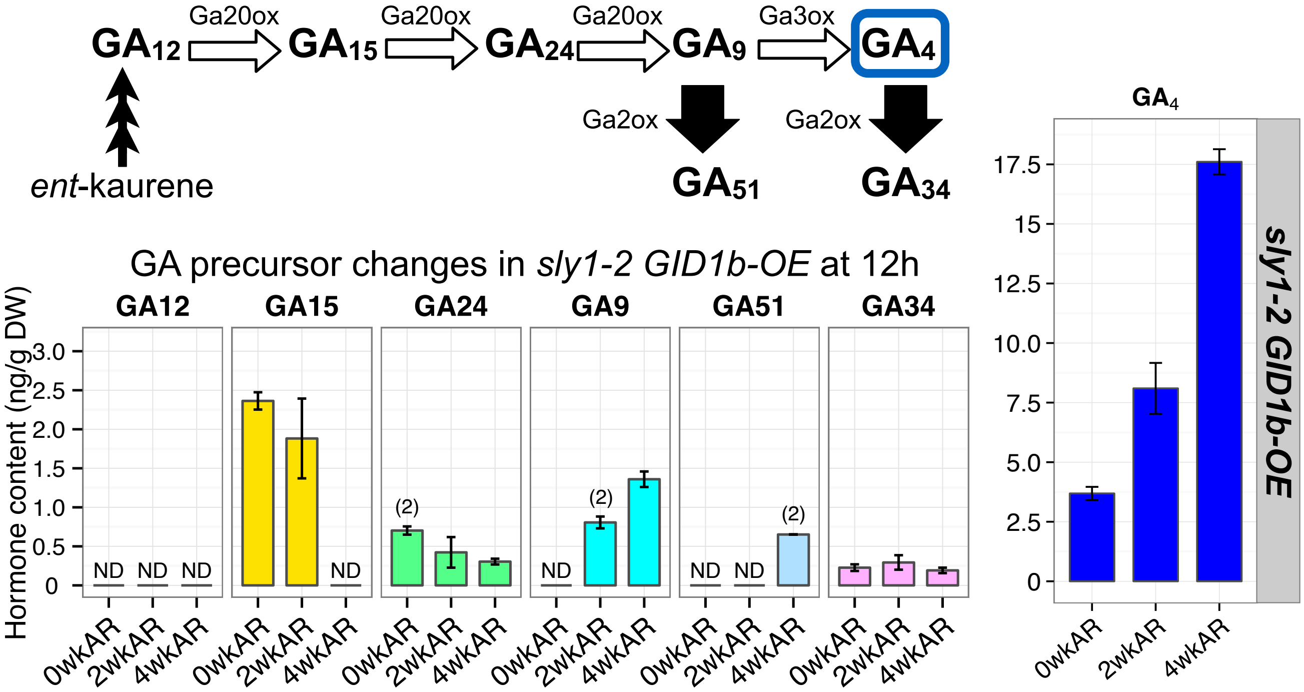

Supplementary Figure 13. GA precursor and catabolite measurements for 12h imbibed *sly1-2 GID1b-OE* at 0, 2, and 4 weeks of after-ripening (0, 2, 4wkAR). Bioactive GA_4 levels are shown for comparison. "(2)" indicates that endogenous levels were only detected twice, and bars without error bars were only detected once of total replicates assayed. Error bars represent SE.

		Early Phase II[a]		Late Phase II[b]	
ID	Gene	logFC	adj.P.Val	logFC	adj.P.Val
***sly1-2* DvsWT[c]**					
At4g02780	CPS1	—		3.26	2.51×10^{-10}
At1g79460	KS1	0.96	1.41×10^{-02}	—	
At5g25900	KO1	—		0.91	7.47×10^{-05}
At2g32440	KAO2	—		0.93	1.83×10^{-05}
At4g25420	GA20ox1	1.75	1.58×10^{-09}	2.59	1.06×10^{-13}
At5g51810	GA20ox2	1.64	1.94×10^{-09}	5.27	1.06×10^{-19}
At5g07200	GA20ox3	0.96	7.00×10^{-04}	4.83	1.52×10^{-16}
At1g15550	GA3ox1	—		0.86	5.67×10^{-04}
At1g80340	GA3ox2	—		-1.53	1.70×10^{-06}
At1g30040	GA2ox2	0.99	7.85×10^{-04}	0.92	5.79×10^{-04}
***sly1-2* ARvsWT[d]**					
At4g02780	CPS1	0.85	1.87×10^{-02}	3.27	5.49×10^{-10}
At1g79460	KS1	0.96	9.54×10^{-03}	—	
At5g25900	KO1	—		1.02	3.05×10^{-05}
At2g32440	KAO2	0.56	6.47×10^{-03}	1.14	1.96×10^{-06}
At4g25420	GA20ox1	1.87	5.79×10^{-10}	2.37	1.56×10^{-12}
At5g51810	GA20ox2	1.65	1.73×10^{-09}	4.06	2.19×10^{-17}
At5g07200	GA20ox3	1.25	1.95×10^{-05}	4.03	1.47×10^{-14}
At1g80340	GA3ox2	—		-1.45	7.12×10^{-06}
At1g80330	GA3ox4	—		1.97	4.44×10^{-09}
At1g30040	GA2ox2	1.04	2.76×10^{-04}	—	
***sly1-2* GIDvsWT[e]**					
At4g02780	CPS1	—		2.93	1.73×10^{-09}
At1g79460	KS1	0.81	4.18×10^{-02}	0.87	1.28×10^{-02}
At5g25900	KO1	0.61	1.03×10^{-02}	1.03	1.40×10^{-05}
At2g32440	KAO2	—		0.69	5.94×10^{-04}
At4g25420	GA20ox1	1.99	1.57×10^{-10}	2.15	3.61×10^{-12}
At5g51810	GA20ox2	1.91	1.27×10^{-10}	4.76	2.40×10^{-19}
At5g07200	GA20ox3	1.24	3.39×10^{-05}	4.34	1.20×10^{-15}
At1g15550	GA3ox1	—		0.72	3.16×10^{-03}
At1g80340	GA3ox2	—		-1.76	1.89×10^{-07}
At1g30040	GA2ox2	—		0.84	1.35×10^{-03}

[a] 0h timepoint
[b] 12h timepoint
[c] *sly1-2*(Dormant) vs L*er* wt, Nelson and Steber, 2017
[d] *sly1-2*(After-ripened) vs L*er* wt, Nelson and Steber, 2017
[e] *sly1-2 GID1b-OE* vs L*er* wt, Nelson and Steber, 2017

Supplementary Figure 14. Microarray reanalysis to identify GA biosynthetic and catabolic genes that are differentially regulated in *sly1-2* background lines relative to L*er* wt. Original datasets from Nelson and Steber (2017).

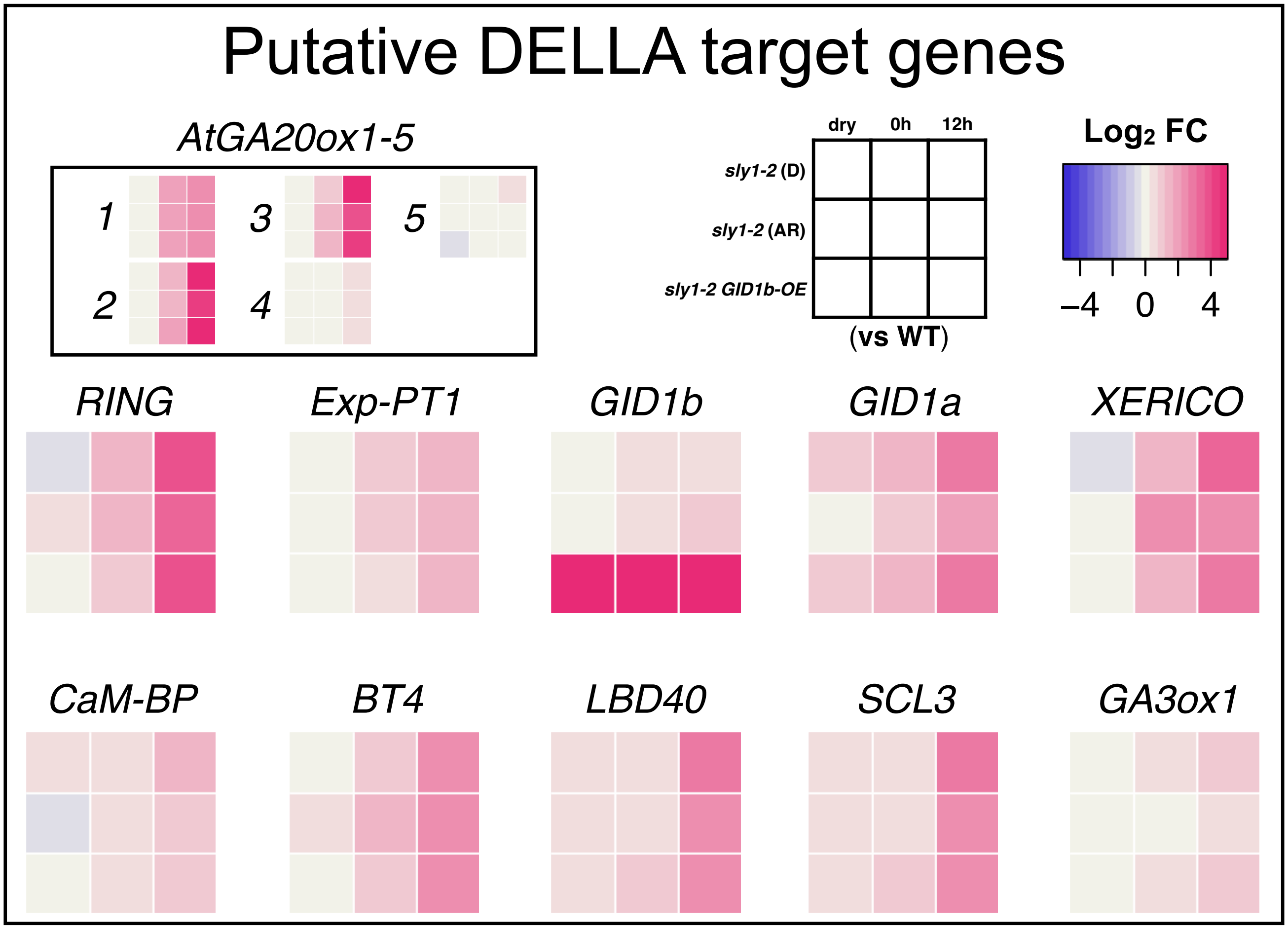

Supplementary Figure 15. Reanalysis of transcript levels of previously identified DELLA-regulated genes (Zentella et al., 2007) to determine if they are regulated by *sly1-2*, after-ripening or *GID1b* overexpression. Transcript levels are shown at dry, 0h, and 12h imbibition timepoints in dormant (D) *sly1-2*, 19 mo after-ripened (AR) *sly1-2*, and in *sly1-2 GID1b-OE* in heat maps relative to levels in L*er* wt (WT) at 2 wkAR. Magenta indicates a positive and blue indicates a negative fold change (FC) relative to WT. Data come from the reanalysis of microarray datasets from Nelson and Steber (2017) and Nelson et al. (2017). Previous reports did not compare dry and imbibed seed transcript levels. Gene identifiers for genes shown: At4g19700 (RING), At2g45900 (Exp-PT1), At3g63010 (AtGID1b), At3g05120 (AtGID1a), At2g04240 (XERICO), At3g52870 (CaM-BP), At5g67480 (BT4), At1g67100 (LBD40), At1g50420 (SCL3), At1g15550 (GA3ox1), At4g25420 (GA20ox1), At5g51810 (GA20ox2), At5g07200 (GA20ox3), At1g60980 (GA20ox4), At1g60980 (GA20ox5).
